# Sparcle: assigning transcripts to cells in multiplexed images

**DOI:** 10.1093/bioadv/vbac048

**Published:** 2022-06-17

**Authors:** Sandhya Prabhakaran

**Affiliations:** Computational and Systems Biology Program, Sloan Kettering Institute, Memorial Sloan Kettering Cancer Center, New York, NY 10065, USA

## Abstract

**Motivation:**

Imaging-based spatial transcriptomics has the power to reveal patterns of single-cell gene expression by detecting mRNA transcripts as individually resolved spots in multiplexed images. However, molecular quantification has been severely limited by the computational challenges of segmenting poorly outlined, overlapping cells and of overcoming technical noise; the majority of transcripts are routinely discarded because they fall outside the segmentation boundaries. This lost information leads to less accurate gene count matrices and weakens downstream analyses, such as cell type or gene program identification.

**Results:**

Here, we present Sparcle, a probabilistic model that reassigns transcripts to cells based on gene covariation patterns and incorporates spatial features such as distance to nucleus. We demonstrate its utility on both multiplexed error-robust fluorescence *in situ* hybridization, single-molecule FISH data, probabilistic cell typing *in situ* sequencing, spatially resolved transcript amplicon readout mapping and MERFISH from Vizgen. Sparcle improves transcript assignment, providing more realistic per-cell quantification of each gene, better delineation of cell boundaries and improved cluster assignments. Critically, our approach does not require an accurate segmentation and is agnostic to technological platform.

**Availability and implementation:**

The code is available at: https://github.com/sandhya212/Sparcle_for_spot_reassignments

**Contact:**

sandhya.prabhakaran@moffitt.org

**Supplementary information:**

Supplementary data are available at *Bioinformatics Advances* online.

## 1 Introduction

Imaging-based spatial transcriptomics comprises a set of ground-breaking technologies that scale up the number of genes that can be quantified within tissues (Marx, 2021). The emergence of these approaches has made it possible to resolve cell-type heterogeneity and to gain a holistic understanding of gene expression within spatial context (Allen Brain Map, 2019; Moffitt *et al.*, 2018). However, computational tools to interpret these data are severely lacking. A fundamental challenge is the construction of a biologically accurate cell-by-gene count matrix, which requires cell segmentation as a first step. Differences in cell size and morphology, occlusion, overlapping cells, de-nucleated cells and technical noise from the imaging instruments make simultaneous automated segmentation of multiple cell types extremely difficult.

The boundaries of nuclei are easier to resolve than cell outlines. Current automated cell segmentation methods identify nuclei using data from a nuclear marker channel such as DAPI, then radially dilate these borders by a few pixels to approximate cell boundaries. This tends to leave a substantial fraction of ‘dangling’ transcripts outside of defined cell boundaries (Fig. 1.1). Dangling transcripts are potentially assignable to multiple cells, especially as distance from the nucleus increases, and are thus discarded prior to generating the count matrix. Moreover, 2D images only reveal a slice of the cell volume. The transcripts present in peri-nuclear sections therefore represent a very sparse sampling of the cellular transcriptome, which underpowers downstream analyses of cell types and gene programs.

**Fig. 1.**
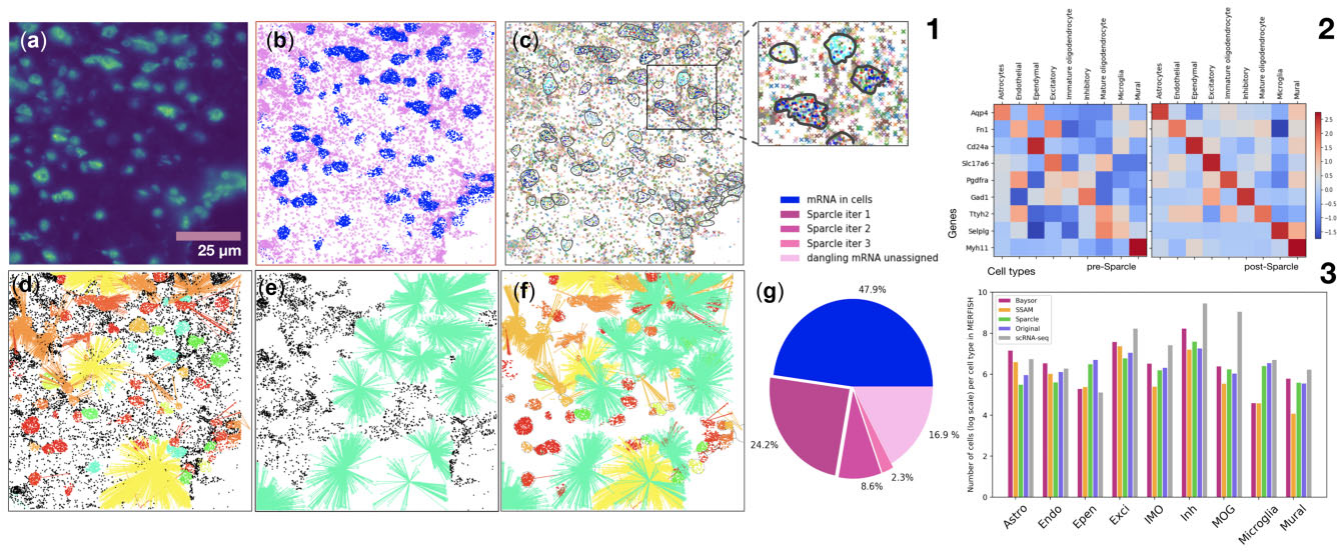
Sparcle captures transcriptomic diversity that is present in spatial transcriptomic images but is discarded by typical analysis. (1: **a–f**) A single 2D exemplar FoV of the mouse hypothalamic posterior optic region imaged by MERFISH (Moffitt et al., 2018). (a) MERFISH image showing the DAPI marker identifying cells. Since DNA is not uniformly distributed throughout the nucleus, there are regions of enriched DNA density that are seen as bright spots. (b) Conservative segmentation captures n = 12,000 transcripts (dots; ∼48% of mRNAs) (Moffitt *et al.*, 2018) leaving ∼14 000 ‘dangling’ transcripts (crosses). Current methods fail to accurately segment neuronal cell morphologies in particular, which often contain transcripts far from the cell body. (c) Spatial diversity of transcripts within (dots) and outside (crosses) the cell segmentation. Each mRNA is colored according to corresponding gene (*n* = 140 genes assayed). Inset, magnification highlighting the diversity of dangling transcripts; solid lines indicate segmentation boundaries. (d–f) For the FoV shown in (a), Sparcle captures 27%, 10.3% and 1.2% of total mRNAs in iteration 1 (d), iteration 2 (e) and iteration 3 (f), respectively. [Panel (f) also overlays all the mRNA assignments from previous iterations.] (f) Overall dangling mRNA assignments across the three iterations, with unassigned dangling mRNAs removed. Dangling mRNAs assigned to one cluster are connected to the cell centroid by lines of the cluster color; dots are unassigned dangling mRNAs. (g) Fraction of all mRNAs recovered across 400 MERFISH FoVs by segmentation and Sparcle assignment. (**2**) Sparcle improves the cell-type specificity of canonical gene assignment for most cell types. Z-score-normalized gene expression (rows) is averaged over vectors per cluster representing cell type (columns). (**3**) Application benchmarking showing comparison of Sparcle to the published results in SSAM [Park *et al.*, 2021] and Baysor [Petukhov *et al.*, 2022] for individual cell types in MERFISH, along with matching scRNA-seq data. Figure shows the number of cells (in log scale) for each of the cell types across the three methods (Baysor, SSAM and Sparcle) alongside the original data [shown as ‘Original’] as provided in (2) for 80 FoVs (= 1 slice of data) and the matching single-cell RNAseq data

To address these issues, we introduce Sparcle (spatial reassignment of spots to cells via maximum likelihood estimation), a comprehensive probabilistic model that assigns dangling transcripts on the basis of gene covariance, as well as the transcripts’ distance between neighboring cells and adjacent transcripts. These patterns include neighboring cells (defined by Euclidean distances between cell centroids), and neighboring transcripts for each cell. Sparcle generalizes to any single-molecule FISH (smFISH) imaging methodology, and we demonstrate its utility on multiplexed error-robust fluorescence *in situ* hybridization (MERFISH; Moffitt *et al.*, 2018), smFISH (Allen Brain Map, 2019), probabilistic cell typing *in situ* sequencing (pciSeq; Qian *et al.*, 2020), spatially resolved transcript amplicon readout mapping (STARmap; Wang *et al.*, 2018) and MERFISH from Vizgen (2021). Images from these datasets pertain to various brain regions where the cell morphology is non-standard, and cells are not particularly densely packed. Sparcle is one of the first methods to capture dangling transcripts; we show that it recovers more than 50% of these mRNAs and validate its performance using scRNA-seq data.

## 2 Sparcle model

Sparcle is a probabilistic model that iteratively assigns dangling transcripts to neighboring cells using maximum likelihood estimation (Supplementary Methods S1.1). Importantly, our approach does not require a highly accurate segmentation as input.

As a first step, the gene expression profiles of the segmented cells are clustered to identify cell types. The large number of transcripts in each cell justifies modeling gene expression as a multivariate Gaussian distribution, based on the central limit theorem assumption. An image can thus be considered as a mixture of cell-specific Gaussian distributions, which can be posed in a Dirichlet process mixture model (DPMM; Neal, 2000) setting to identify heterogeneous cell types. In Sparcle, the user can choose either the DPMM or Phenograph (Levine *et al.*, 2015) for clustering; neither require that cluster numbers are known *a priori*. We then compute a multivariate Gaussian distribution representing gene–gene covariation relationships for each cluster in our data by learning the cluster-specific first- and second-order moments (mean and covariance) of each cluster. For every dangling mRNA, the algorithm constructs a circular weighted ‘mock’ cell with radius r, centered at the mRNA, which captures proximal mRNA neighbors (Supplementary Fig. S5 and Methods S1.2–S1.4). The contribution of each gene to the mock cell is inversely weighted by its distance from the dangling mRNA. Weighting the mock cell enables capturing the non-uniform distribution of transcripts in the nuclear region versus regions away from the cell body. Next, Sparcle generates a maximum likelihood estimate (MLE) of which cluster (often corresponding to cell type) the mock cell most likely represents and assigns the dangling mRNA to the cell with that cluster label nearest to the mock cell, and updates the count matrix (Supplementary Methods S1.5 and S1.6). This process of assigning dangling mRNAs continues for a fixed number of iterations by updating and clustering the count matrix, recalculating the cluster-specific moments and revising the cellular assignments to clusters at each iteration. With a user-provided cell segmentation and a minimal set of default parameters, Sparcle can robustly assign dangling mRNAs in under 10 min on a high-performance cluster for a typical MERFISH field of view (FoV) consisting of ∼80 cells and ∼14 000 dangling mRNAs. The outputs of the iterative model are (i) a more realistic count matrix, based on improved mRNA assignments to cells, (ii) cluster assignments for each cell and (iii) improved delineation of cell boundaries. We provide a graphical abstract of Sparcle in Supplementary Figure S1.

## 3 Results

### 3.1 Sparcle increases the fraction of total transcripts assigned to cells in MERFISH images

The mammalian brain is characterized by diverse cell types with complicated morphologies and cellular interactions that can greatly benefit from multiplexed imaging and spatial analysis. A MERFISH image of the mouse hypothalamus posterior optic region comprises ∼30 000 cells from 70 different neuronal populations and 140 assayed genes (Moffitt *et al.*, 2018). From this image, 400 smaller FoVs were imaged at high magnification (Fig. 1.1a); segmentation using dilated cell nuclei revealed that ∼52% of mRNAs, which exhibit substantial spatial heterogeneity, remain unassigned (Fig. 1.1b and c). Using these individual FoVs (Fig. 1.1d–f), Sparcle increases the fraction of total transcripts that are assigned to cells by assigning 24.2%, 8.6% and 2.3% out of the total transcripts in iterations 1, 2 and 3, respectively (Fig. 1g), ultimately capturing 68% of the 2.8 million dangling transcripts in this full dataset.

### 3.2 Evaluating Sparcle using matching scRNA-seq data

To evaluate the cell populations identified by Sparcle, we used paired scRNA-seq data (31 299 cells) as ground truth, considering a subset of 900 genes containing MERFISH probes and neuronal genes, as utilized in Moffitt *et al.* (2018), to call cell types. We used Phenograph (Levine *et al.*, 2015) to cluster both the scRNA-seq and MERFISH count matrices before and after applying Sparcle. Post-Sparcle, we observe that the cell-type proportions are more concordant with those of the scRNA-seq data (Supplementary Fig. S2a-i and ii).

To further assess Sparcle’s performance, we tested the similarity of count matrices derived from MERFISH and matching scRNA-seq using two different covariance approaches (Supplementary Methods S1.8 and S1.9). The Gramian (cluster–cluster) covariance matrices from scRNA-seq and MERFISH are more similar after Sparcle (*P* = 0.620, Box’s *M* test; Frobenius norm = 2.34) than before Sparcle (*P* = 0.309; Frobenius norm = 15.18; larger *P*-values and smaller norms indicate more similar matrices), showing that Sparcle assignments improve MERFISH cell-ype calls relative to ground truth (Supplementary Fig. S2b). The cluster–cluster cross-covariance matrix between scRNA-seq and post-Sparcle MERFISH exhibits stronger variances within clusters (dominant diagonal) than between clusters, compared with the pre-Sparcle cross-covariance matrix, especially for non-neuronal cell types (Supplementary Fig. S2c). This covariance pattern signals stronger relationships within cell types and weaker relationships between different cell types, supporting the accuracy of Sparcle assignments.

Finally, Sparcle clearly improves the assignment of canonical cell-type-specific genes for most clusters, including excitatory neuronal, endothelial, ependymal and astrocyte cells (Fig. 1.2). This observation supports the validity of the higher proportions of these cell types that result from Sparcle transcript assignments (Supplementary Fig. S2a-ii).

### 3.3 Sparcle captures more information in MERFISH-derived cell clusters

We sought to determine whether Sparcle assignments capture more transcriptional variability and lend greater structure to molecular quantification data from imaging datasets. To do this, we examined pairwise Pearson correlation coefficients between cluster expression profiles extracted from the mouse hypothalamus scRNA-seq and MERFISH data, both before and after Sparcle, and used Loewner ordering to compare matrices (see Supplementary Methods Section S1.10).

We first extracted and examined two broad neuronal cell types. We built a pre-Sparcle MERFISH matrix with 100 randomly sampled excitatory cells stacked over 100 inhibitory cells and a post-Sparcle matrix with the same cells, then computed Pearson correlations with a similarly constructed scRNA-seq matrix of 100 random cells per cell type (Supplementary Fig. S3a). The matrices reveal higher scRNA-seq correlations and greater correlation structure for both cell types after using Sparcle (Supplementary Fig. S3a, right). Notably, Sparcle assignments substantially improve the recovery of excitatory cells, which only number approximately half of the inhibitory cells. Furthermore, Loewner ordering reveals that the excitatory block in the post-Sparcle correlation matrix captures additional variability (subtracting this block in the post-Sparcle matrix from the pre-Sparcle matrix generates a positive semi-definite matrix, whereas subtraction in the other direction does not).

For non-neuronal clusters, we placed astrocytes, endothelial and ependymal cells in one set (NN set 1) and immature and mature oligodendrocytes, microglia and mural cells in another (NN set 2), and performed Pearson correlation and Loewner ordering (Supplementary Fig. S3b). Both cell sets show clearly higher correlations with scRNA-seq, and the Pearson correlation block diagonals in the post-Sparcle matrix (Supplementary Fig. S3b, right) indicate stronger relationships. Loewner ordering of the individual blocks likewise indicates that dangling transcript assignment captures more variability post-Sparcle. This additional variability can be exploited to refine cell-type assignment and improve gene-level analyses.

### 3.4 Sparcle generalizes to additional imaging modalities

To test the generalizability of Sparcle, we examined public smFISH (Allen Brain Map, 2019), pciSeq (Qian *et al.*, 2020), STARmap (Wang *et al.*, 2018) and Vizgen (2021) images. Additional information about data availability and matching scRNA-seq are provided in the Supplementary Section S2. The Sparcle run on Vizgen is discussed in Supplementary Methods S1.11.

#### 3.4.1 smFISH of mouse primary visual cortex

We use the smFISH images of the adult mouse primary visual cortex, comprising 3500 cells and 1 074 000 mRNA spots from 22 unique genes (Allen Brain Map, 2019) from which we subsampled 250 000 mRNAs [154 000 within cells (61.8%) and 95 000 dangling (38.2%)]. For comparison purposes, we used matching scRNA-seq data comprising 43 498 cells and 727 highly variable genes, including the 22 smFISH genes. We clustered count matrices and annotated smFISH cells and clusters (both before and after Sparcle) based on the four cell types identified by scRNA-seq (Supplementary Figs. S4a and S7). Sparcle recovers 32.2% of the total mRNA across three iterations (26.3% in iteration 1, 4.7% in iteration 2 and 1.2% in iteration 3; Supplementary Fig. S8). Analysis of Gramian matrices reveals that post-Sparcle cell-type covariances are more similar to the matching scRNA-seq cluster covariances (Box’s *M* test, *P* > 0.05; Frobenius norm closer to 0; Supplementary Fig. S4b) and the cluster–cluster cross-covariance matrix between scRNA-seq and MERFISH has a stronger diagonal matrix post-Sparcle (Supplementary Fig. S4c). Cluster analysis shows that Sparcle assigns multiple genes to at least one additional biologically relevant cell type; for example, Sv2C and Thsd7a belong to either endothelial or excitatory cells pre-Sparcle, and expand to both cell types after Sparcle (Supplementary Fig. S4d). We perform an additional experiment using Sparcle based on finer cell-type resolutions (Supplementary Fig. S12). The cluster–cluster cross-covariance post-Sparcle matrix between scRNA-seq and smFISH (Supplementary Fig. S12b) indicates that Sparcle improves cell types (for e, g. Lamp5, Sst, L4IT1, Oligo/VLMC). The cluster analysis in the post-Sparcle setting (Supplementary Fig. S12d) shows that RorB and Cux2-expressed excitatory cells retain their individual clusters and that Cux2 is better refined for L2/3 IT 1 whereas in the pre-Sparcle setting (Supplementary Fig. S12c), Cux2 was spread across L2/3 IT 1, Oligo/VLMC and L4 IT 1.

#### 3.4.2 pciSeq ISS CA1 of mouse hippocampus data

We use the CA1DapiBoundaries_4-3_right.tif (Qian *et al.*, 2020) that had 2024 cells and 92 genes. There were 72 000 mRNA in total of which 11 000 were identified as dangling mRNA. Dimensions of the paired single-cell RNA seq data are 5100 cells and 27 998 genes. We provide the cluster–cluster cross-covariance matrices between published scRNA-seq (Qian *et al.*, 2020) and ISS clusters pre- and post-Sparcle (Supplementary Fig. S9) and observe a clearer separation of interneuron-selective (IS) cell types (IS1, IS3 and IS2) as well as trilaminar, radiatum retrohip and Cck+ (Cck Cxcl14+) clusters post-Sparcle (Supplementary Fig. S9b).

#### 3.4.3 STARmap mouse visual cortex

Sparcle generalizes to STARmap (Wang *et al.*, 2018) 160-gene light data of the mouse visual cortex. In Supplementary Figure S10, we show the expression of canonical STARmap genes (rows) within each cluster (columns) pre- (Section 3.4.1) and post-Sparcle (Section 3.4.2), arranged by hierarchical clustering of the pre- and post-Sparcle matrices, respectively. Sparcle generates clearer blocks of gene expression per cluster and improves assignments for all the cell types. Comparing the cluster–cluster cross-covariance matrices between published scRNA-seq and STARmap clusters pre- (Section 3.4.3) and post-Sparcle (Section 3.4.4) indicate stronger within-cluster variances (dominant diagonal) post-Sparcle including a clearer separation of inhibitory neuronal cell types (Vip, Sst, Pvalb) without any mixing with excitatory neuronal clusters (eL2/3, eL4, eL5 and eL6) post-Sparcle.

## 4 Discussion

Sparcle addresses an important need in the rapidly evolving field of single-cell spatial transcriptomic imaging—the recovery of transcripts outside of segmentation cell boundaries. Our approach assigns dangling transcripts to cells based on their gene expression similarity, through an imaging-platform-independent and comprehensive probabilistic model.

Sparcle takes a conservative approach to dangling mRNA assignment since there is no ground truth for cell segmentation, and many cell morphologies are very complex; for example, neuronal cell bodies are typically partially segmented, whereas mRNA-bearing axons are not usually segmented at all. On MERFISH and smFISH images of brain sections, Sparcle recovers more than 50% of dangling mRNAs, driving improved cell-type calls and gene-level analyses. We have shown that Sparcle mRNA assignments lead to refined count matrices and improved assessment of cell-type heterogeneity.

Spot-based Spatial cell-type Analysis by Multidimensional mRNA density estimation (SSAM; Park *et al.*, 2021) and Bayesian Segmentation of Spatial Transcriptomics Data (Baysor; Petukhov *et al.*, 2022) are recent quantification approaches that aim to circumvent the need for cell segmentation. Figure 1.3 shows the comparison of Sparcle to the published results in Baysor and SSAM for individual cell types in MERFISH, alongside the original data [shown as ‘Original’ (purple bars) as provided in Moffitt *et al.* (2018)] for 80 FoVs (= 1 slice of data) and matching scRNA-seq data (shown as gray bars). We note that although the absolute numbers of cells recovered differ across these methods, which can be attributed to image pre-processing and subsequent analysis by each method, the proportion of cells recovered per cell type through Sparcle is either lesser or at par the ‘Original’ data and this can be attributed to Sparcle’s conservative assignment of mRNAs to cells.

Transcript assignment by Sparcle can be further improved. Sparcle currently utilizes an MLE protocol, which does not assume an informative prior distribution for the dangling mRNAs. For any given gene, we can introduce a prior distribution for the localization of its transcripts and implement a maximum *a posteriori* Bayesian inference procedure based on their spatial diversity. Sparcle can also be amended to work with an initial set of known cell-type distributions for example those cell types estimated from paired single-cell RNA sequencing data. Another enhancement to Sparcle would be the refinement of cell-type morphologies and cell neighborhoods based on the rescued dangling transcripts. To cater to heterogeneous cell morphologies, Sparcle can be fed refined cell segments generated through specialized cell morphology-based segmentation methods. Additional improvements can be achieved by staining for cellular membranes and cell bodies (such as PolyA) in addition to nuclei, to provide rough boundaries for entire cells. This improved segmentation would not only help identify latent cells (due to missing nuclear markers), multinucleated cells and doublets (due to overlapping cells) but also aid to resolve dangling transcripts positioned farther from the nucleus.

Furthermore, a software version in a compiled language such as Java or C++ may also run more efficiently. Sparcle could further be extended to 3D by building a spherical mock cell and operating in a 3D Euclidean coordinate system without changing the logic of the underlying algorithm. With these improvements, we would be able to further generalize Sparcle on densely packed tissues to understand the distribution and recovery of dangling mRNAs to aid clearer demarcation of cell neighborhoods and cell-type regions.

Sparcle relies on imperfect region-independent cell segmentations as a starting point and uses MLE to assign transcripts to inferred cell clusters. Given the high number of dangling mRNAs, this is a relatively simple strategy that does not require customization for different imaging protocols and can be easily parallelized across FoVs or dangling mRNAs (Supplementary MethodsS1.7). Sparcle is shown to effectively assign dangling mRNAs to cells and correct the count matrices. We hope that the computational infrastructure provided by Sparcle will further contribute to the emerging landscape of high-throughput spatial transcriptomic image analysis.

## Supplementary Material

vbac048_Supplementary_Data

## Data Availability

Links to the datasets used in this article are available in Section 2 (Availability of data and materials) of the online supplementary material.
