# Supplementary material for: Sparcle: assigning transcripts to cells in multiplexed images
Source: Bioinform Adv. 2022 Jun 17;2(1):vbac048. doi: 10.1093/bioadv/vbac048 (PMC9710569; doi:10.1093/bioadv/vbac048)
Supplement: vbac048_Supplementary_Data [file vbac048_supplementary_data.zip › BioinformaticsAdvances_Sparcle_Supplementary.docx]

**Supplementary for Sparcle: assigning transcripts to cells in multiplexed images**

**Author:** Sandhya Prabhakaran^1,2^

^1^Computational and Systems Biology Program, Sloan Kettering Institute, Memorial Sloan Kettering Cancer Center, New York, NY, 10065, USA

^2^Current address: Department of Integrated Mathematical Oncology, Moffitt Cancer Center and Research Institute, Tampa, FL, 33612, USA

Corresponding author: [sandhya.prabhakaran@moffitt.org](mailto:sandhya.prabhakaran@moffitt.org)

Abstract

Background

Imaging-based spatial transcriptomics has the power to reveal patterns of single-cell gene expression by detecting mRNA transcripts as individually resolved spots in multiplexed images. However, molecular quantification has been severely limited by the computational challenges of segmenting poorly outlined, overlapping cells, and of overcoming technical noise; the majority of transcripts are routinely discarded because they fall outside the segmentation boundaries. This lost information leads to less accurate gene count matrices and weakens downstream analyses, such as cell type or gene program identification.

Results

Here, we present Sparcle, a probabilistic model that reassigns transcripts to cells based on gene covariation patterns and incorporates spatial features such as distance to nucleus. We demonstrate its utility on both multiplexed error-robust fluorescence in situ hybridization in situ hybridization (MERFISH), single-molecule FISH (smFISH) data, probabilistic cell typing in situ sequencing (pciSeq), spatially-resolved transcript amplicon readout mapping (STARmap) and MERFISH from Vizgen.

Conclusions

Sparcle improves transcript assignment, providing more realistic per-cell quantification of each gene, better delineation of cell boundaries, and improved cluster assignments. Critically, our approach does not require an accurate segmentation and is agnostic to technological platform.

**Keywords**

Image analysis, Bioinformatics, Data analysis, Spatial transcriptomics, SPARCLE

1. Methods

1.1 Algorithmic overview

As input, Sparcle takes approximate cell segmentations generated using any method for a Field of view (FoV). The cell segments across all the FoVs are collected, and Sparcle creates a global count matrix of cells and genes, which is clustered to identify cell types. The large number of transcripts in each cell justifies modelling gene expression as a Gaussian distribution, based on the central limit theorem assumption. An image can thus be considered as a mixture of cell-specific Gaussian distributions, which can be used in a Dirichlet process mixture model (DPMM) (13, 14) for identifying heterogeneous cell types. First, Sparcle learns the cluster-specific first and second order moments (mean and covariance) of each cluster. Next, for each dangling mRNA, a mock cell is created using mRNA transcripts occurring within a certain radius of the dangling mRNA. The mock cell is compared to previously computed cluster-specific moments with the objective of maximizing the likelihood of a dangling mRNA being similar to a cluster and then assigning the mRNA to the closest neighboring cell belonging to the most similar cluster. Neighboring cells are calculated based on Euclidean distances between the mRNA and cell centroids. As each mRNA gets assigned to a cell, we update the count matrix. At the onset of each subsequent iteration, the updated global count matrix is re-clustered, the cluster-specific moments are recomputed and the assignment of dangling mRNAs continues. Through this construction, we are, in essence, building a global model with global cluster-specific means and covariances that all the FoVs have contributed to and will therefore adhere to. Using this global approach, we can ensure that the underlying probabilistic model is constant across FoVs per iteration as well as across iterations.

If we cluster each FoV separately in order to build a local structure, it becomes necessary to ‘match’ the clusters between FoVs. This can be both computationally intensive and unstable, because not all cell types are present in all FoVs, and thus, clusters can differ across FoVs. To build the global structure, we need to perform roughly 400 x k x k’ pairwise stable matching of clusters between FoVs, where k and k’ are the number of clusters for any given pair of FoVs. These comparisons would be riddled by missing or unseen clusters, leading to an improper global structure.

Sparcle therefore resorts to constructing a global structure of cell types from the beginning, to ensure a reliable set of clusters to which dangling mRNAs can be assigned. Sparcle comprises several canonical building blocks, including clustering to identify cluster-specific moments, constructing a mock cell for each dangling mRNA to enable comparison of a dangling mRNA to cluster moments, and maximum likelihood estimation.

1.2 Mockcell window size and shape choice

The mockcell is designed as a circle centered at the dangling mRNA. We consider a circular window to approximate the cell body of an average neuronal cell. To estimate the approximate radii of two mockcells A and B, we draw bounding boxes around those cells (see **Fig. S5, S6** for example corresponding to calculations below). The area of a cell’s bounding box = $( x_{1}- x_{2})*\left( y_{1}-y_{2} \right)$. We equate this to the area of a circle $=\pi r^{2}$, to estimate $r$.

Area of Cell body A = (636-567) * (1200-1113) $=\pi r^{2}$

→ $r$ = 44 pixels

Area of Cell body B = (1548-1404) * (1569-1461) $=\pi r^{2}$

→ $r$ = 70 pixels

Since most cell bodies in MERFISH and smFISH data have $r$ <= 75 pixels, we have set $r$ = 80 pixels as the default radius for a mockcell’s cell body. Cell segmentation algorithms relying on DAPI and/or PolyA stains typically only segment the cell body and tend to lose the neuronal processes. To account for a cell’s neuronal processes and thereby further refine cell types, we increase the radius of a mockcell to 150 pixels to scan farther neighborhoods for potential mRNA patterns found in the processes pertaining to each of the cell types, and thereby their morphologies.

1.3 Weighted mockcell creation

Every dangling mRNA is represented as a mockcell and is a vector of length equal to the number of genes, $G$. Using the circular window of radius $r$ centered at the dangling mRNA, a mockcell is constructed using an inverse distance weighting (IDW) method where distances from the dangling mRNA (or the center) to each of the neighboring mRNA falling within $r$ are interpolated to create a weighted average for each $g in G$. With this construction, mRNAs closest to the dangling mRNA have more influence than those farther away. The weighted average $u_{g}$ for each gene $g$ for the mockcell centered at $x$ is calculated based on the simplest weighting function or Shepard’s method (21) that uses the weights in inverse power as:

$u_{g}\left( x \right)=\left( \frac{\sum_{i=1}^{N} w_{i}\left( x \right)u_{i}}{\sum_{i=1}^{N} w_{i}\left( x \right)} \right)^{2}$when $d\left( x,x_{i} \right)!=0$

and

$w_{i}\left( x \right)=\left( \frac{1}{d\left( x,x_{i} \right)} \right)^{2}$where $d\left( x,x_{I} \right)$ is the distance between the interest point $x$ and a point in the neighborhood $x_{i}$ (denoting a neighboring mRNA) where there are $i=1,\ldots N$ observations of gene $g$ in the neighborhood and $w_{i}\left( x \right)$ is the weight or predicted value at point $x$.

We use a modification of Shepard’s method that calculates the interpolated value using only nearest neighbors within the r-sphere (instead of the entire sample space). Weights in the above equation are modified in this case to give $w_{i(modified)}\left( x \right)=\left( \frac{\max\left( 0,r-d\left( x,x_{i} \right) \right)}{r*d\left( x,x_{i} \right)} \right)^{2}$that are used to compute the modified weighted average $u_{g\_mod}(x)$ which is entered for each $g$ the mRNA accounts for in the mockcell.

1.4 Spatial distances and how they are incorporated into Sparcle

By orienting every image to a 2D Cartesian coordinate system, we assume each image to reside on a Euclidean plane. This allows us to calculate the inter-cellular distances using pairwise Euclidean distances between cell centroids. Further, the local concentration of mRNA transcripts within a mockcell is derived by computing the Euclidean distances between the transcripts and the mockcell’s centroid.

1.5 Maximum likelihood method

We assume that the gene expression in each cell follows a multivariate Gaussian distribution. This enables us to view an image as a mixture of cell-specific Gaussian distributions, and to use a Dirichlet process mixture model (DPMM) for clustering the cells into  $K$ different cell types.

In order to assign a dangling mRNA, $m$, to one of the inferred cell types $k=1\ldots K$, we use the maximum likelihood (ML) method. Assuming there are $k$ cell types, the mockcell originating at a dangling mRNA, is tested for each cell type’s parameters i.e. multivariate mean $\mu_{k}$ and covariance $\Sigma_{k}$ to identify $k_{ML}$ that maximizes the likelihood distribution. We assume the likelihood distribution to be that of a multivariate Gaussian. The analytic form of the ML method can be written as:

$\underset{\theta\in\Theta}{\hat{\theta}_{\mathrm{ML}}= arg max} p(m|\mu_{k}, \Sigma_{k})$ and

$$p\left( m | \mu_{k}, \Sigma_{k} \right)=\frac{exp(-0.5 \left( m-\mu_{k} \right)^{T}\Sigma_{k}^{-1} \left( m-\mu_{k} \right))}{\sqrt{{(2\pi)}^{d}-|\Sigma_{k}|}}$$

where $\hat{\theta}_{\mathrm{ML}}$ is the maximum likelihood estimate of the Gaussian parameters $\theta=\left[ \mu_{k}, \Sigma_{k} \right]$, $\Theta$ is the finite-dimensional parameter space for $\theta$, $d$ is the number of genes and $p\left( m | \theta\right)$ is the non-degenerate case of the multivariate Gaussian probability density function (pdf). We ensure the non-degenerate pdf by constructing positive (semi) definite $\Sigma_{k}$using Givens rotations as described in the next section. The dangling mRNA is assigned to the nearest neighboring cell belonging to the closest ‘likely’ cell type, $k_{ML}$ described by $\hat{\theta}_{\mathrm{ML}}$. The count matrix entry for the cell identified and gene associated by the dangling mRNA is updated. The assigned dangling mRNA is removed from the candidate list of mRNAs to be assigned. The ML method is known to be optimal for large data sizes (22).

1.6 Givens rotations on the covariance matrices

To ensure that covariance matrices used in the maximum likelihood density estimation are positive semi-definite, and to avoid unnecessary cost overhead by inverting these matrices, we perform QR decomposition using the Givens rotations on the covariance matrices (23). These are efficient transformations on sparse matrices and can be better parallelized as well.

For any real square matrix $A$, the QR decomposition is $A=QR$ where *Q* is an orthogonal matrix and *R* is an upper triangular matrix. QR decompositions are computed with a series of Givens rotations. In each rotation an element in the subdiagonal of the matrix is zeroed out, to form the symmetric R matrix. The product of all the Givens rotations forms the orthogonal Q matrix.

In practice, Givens rotations are not actually carried out by building the entire matrix to perform the matrix multiplication between $Q$ and $R$. Instead, only the upper triangle of $R$ is built out and multiplied with $Q$ during each Givens rotation which is equivalent to a sparse Givens matrix multiplication, without having to explicitly handle the sparse elements. This Givens rotation procedure is useful when only a relatively few off diagonal elements need to be zeroed out implying the matrix is still dense, and can be more easily parallelized ([24](#_ENREF_24), [25](#_ENREF_25)).

1.7 Runtime complexity analysis

We calculate the Big O notation for an entire run of Sparcle for one MERFISH FoV. On average, a MERFISH FoV consists of 80 cells, of which ~50% of mRNA transcripts fall within a cell and the rest are dangling mRNA. We consider $n$ the number of cells, $d$ the number of genes, $m$ the number of dangling mRNA transcripts in the first iteration, $m'$ the number of dangling mRNA transcripts in the subsequent iterations,$x$ the number of line segments constituting each cell boundary, $k$ the number of mRNA neighbors per dangling mRNA for constructing the mockcell and $c$ the total number of iterations. We have observed that setting $c$ to 3 is usually sufficient since most of the dangling mRNAs are assigned in iterations *c* = 1 and 2.

Below is the detailed code flow implemented in Sparcle using four main steps with each step having both parallel and non-parallel operations.

1. Step 1:
   1. **processFOV(f):** Each parallel task in processFOV handles one FoV, f
      1. reads in an FOV
      2. calculates the count matrix (cells x genes) using mRNA within the cell and
      3. builds the list of dangling mRNAs.
   2. Consolidate all count matrices to build an overall count matrix, and compute the cluster means and covariances
   3. Runtime: $O(n)$, 1 min
2. Step 2:
   1. **processIter0(f):** First round of mRNA assignments occur here. Each parallel task in processIter0 handles mRNA assignments within an FoV, f
      1. includes a series of projections to identify the cell centroids, cell boundaries, to plot all the cells per FoV
      2. Dangling mRNA assignments to cells using MLE.
         1. Maximum likelihood estimation calculations: ${O(nd}^{2}$)
         2. Euclidean distance to find neighboring cells for cell $i$:$O(n)$
            1. n subtractions for (cell_centroid_i - neigh_cell_centroid)
            2. n squares of (2a)
            3. (n-1) further additions to add (2b)
            4. Final one square root. (So each of these is (at most) linear in $n$, and hence so is the whole algorithm.)
         3. Perform a.ii.2 for $n$ cells = $nO(n)$
         4. Euclidean distance to create j^th^ mock cell using neighboring mRNA for j^th^ dangling mRNA: $O(k)$
            1. k subtractions for (dangling mRNA_centroid_j - neigh_mRNA)
            2. k squares of (4a)
            3. (k-1) further additions to add (4b)
            4. Final one square root. (So each of these is (at most) linear in $k$, and hence so is the whole algorithm.)
         5. Perform a.ii.4 for $m$ cells = $mO(k)$
      3. Updating the count matrix with new assignments
      4. Decrementing the dangling mRNA list by removing those mRNA that were assigned to cells.
   2. Cluster the updated count matrix and update the cluster means and covariances
   3. Runtime: $O({nd}^{2})+nO(n)+mO(k) = O({nd}^{2})$, 3 mins
3. Step 3:
   1. **processItern(f):** ‘*c*’ rounds of mRNA assignments happen here. Each parallel task in processItern handles mRNA assignments within an FoV, f
      1. Dangling mRNA assignments to cells using MLE.
      2. Updating the count matrix with new assignments
      3. Decrementing the dangling mRNA list by removing those mRNA that were assigned to cells.
   2. Runtime $c$: $O({nd}^{2})$, 2.5 mins
   3. Final clustering of the updated count matrix and update the cluster means and covariances
4. Step 4:
   1. **processOverall(f):** parallelize the overall merging and plotting of mRNA assignments per FoV, f
   2. Runtime: $O\left( nm \right)+cO(m)$, 1.5 mins

Therefore, overall runtime complexity is $O(n)+O({nd}^{2})+cO({nd}^{2})+O(nm)+cO(m) \sim$ $O\left( \mathrm{nd}^{2} \right)+ O\left( \mathrm{nm} \right) \sim O\left( nm \right) \sim$ $O(m)$

The runtime is given based on Sparcle runs on an HPC. The HPC specifications were:

- 232 compute cores across 13 compute nodes
- PowerEdge R420 server
- Dual Intel(R) Xeon(R) CPU E5-2450 2.1 GHz CPUs for a total of 16 hyper-threaded cores (32 threads)
- 64 GiB RAM
- 5.3 TB RAID storage with advanced ZFS filesystem
- FreeBSD 11 operating system / CentOS 7 operating system
- Preloaded with multiple open source applications and libraries

1.8 Evaluating cell populations identified by Sparcle using matching scRNA-seq

To further assess Sparcle’s performance, we tested the similarity of count matrices derived from MERFISH and scRNA-seq using two different covariance approaches i.e. cross-covariance matrix (Gramian matrix) and the cluster-cluster cross covariance matrix.

1. The Gramian matrix across clusters for the pre-Sparcle count matrix, post-Sparcle count matrix and matching single-cell RNA-seq data is shown in **Fig. S2b**. For this, we compute the Box’s M test which is a parametric test to check the homogeneity of variance (homoscedasticity). Specifically, it tests for the homogeneity of covariance matrices describing multivariate Gaussian data according to one or more groups. The groups in our setting are pre-Sparcle, post-Sparcle and scRNA-seq. The test compares the product of the log determinants of each of the covariance matrices to the log determinant of the pooled covariance matrix, similar to a likelihood ratio test ([26](#_ENREF_26)). The generated test statistic is called Box’s M statistic and is approximated using a chi-square goodness of fit. The [null hypothesis](https://www.statisticshowto.datasciencecentral.com/probability-and-statistics/null-hypothesis/) for this test is that the observed covariance matrices are equal across groups. This means a non-significant test result (i.e. one with a large p-value) indicates that the covariance matrices are not different ([27](#_ENREF_27), [28](#_ENREF_28)).

The Box’s M test between

- Pre-Sparcle and scRNA-seq: median chi-squared = 0.2556, df = 2, p-value = 0.3091.
- Post-Sparcle and scRNA-seq: median chi-squared = 0.9458, df = 2, p-value = 0.6201.

A p-value greater than 0.05 indicates the variances are homogeneous and higher p-values indicate more homogeneous variances depicting that post-Sparcle and scRNA-seq covariances are more similar. Further, we check the distance between covariances using the Frobenius norm (discussed in the next section).

1. The Cluster-cluster cross covariance matrix between pre-Sparcle clusters with scRNA-seq clusters (Left panel) and between post-Sparcle clusters with scRNA-seq clusters (Right panel) is shown in **Fig. S2c**.

We observe that in the post-Sparcle covariance,

1. the diagonal shows stronger variances **within** clusters than the pre-Sparcle covariance matrix, especially for nonneuronal cell types.
2. the off-diagonal elements indicate *weaker to no* covariance **between** clusters as opposed to the pre-Sparcle and scRNA-seq covariance.

This is a preferred covariance pattern that signals stronger relationships within and weaker relationships between cell types. This means that cell types have garnered relevant mRNA transcripts via Sparcle assignments.

(c) We map canonical genes per cluster for both pre-Sparcle and post-Sparcle cell types in **Fig. 1.2**. We see clear and improved cluster assignments for most of the post-Sparcle clusters.

1.9 Distance between covariance matrices using the Frobenius norm

Consider a m × m covariance matrix $A$ and the set $B$ with all m × m positive definite covariance matrices having similar structure (for example, uniform covariance structure). In order to find the discrepancy between $A$  and the set $B$, we define $D\left( A,B \right)=\min_{\left\{ B^{*}in B \right\}}L\left( A,B \right)$ where $L\left( A,B \right)$  is a measure of the distance between the two m×m matrices  $A$  and $B$. We consider $A$  the covariance matrix from the scRNA-seq measurements and the set $B$ to consist of the pre- and post-Sparcle covariance matrices that originate from the same measurement space. The matrix $B^{*}$ which has the smallest discrepancy can be viewed as that with the most likely or closest to the underlying structure of $A$ . We consider the distance between the two matrices to be the square of the Frobenius-norm, or the F-norm:$L\left( A,B \right)=tr(\left( A-B \right)^{T} \left( A-B \right))$, which is defined as the square of the sum of the absolute squares of its elements ([29](#_ENREF_29)).

For **Fig. S2b**, the Frobenius norm for the Gramian matrices between

- Pre-Sparcle and scRNA-seq is 15.18
- Post-Sparcle and scRNA-seq is 2.34 showing that the post-Sparcle covariance is closer to the scRNA-seq covariance.

1. 10 Loewner ordering of matrices

One way to compare matrices is by using the positive definiteness (pd) or positive-semi definiteness (psd) property. We extract the block matrices from the Pearson’s correlation matrix (**Fig. S3**) and test them for pd or psd using the Cholesky decomposition. Cholesky decomposition factors a psd matrix $A$ into: $A=L L^{T}$ where $L$ is the lower triangular matrix and also the Cholesky factor of $A$. If L is invertible, then *A* is a pd matrix. Since the blocks are from covariance matrices, the blocks satisfy either the pd or psd property. Assume there are two covariance matrices (or multivariate variances) $C$ and $D$ where $C$ and $D$ are by definition pd or psd or verified using the Cholesky decomposition described above. If upon removing $D$ from $C$ (i.e. $C-D$) we get a positive (semi) definite matrix that means that $C$ has captured *more* viable variability within the system and we can write *D <= C*. This way of ordering matrices generates the Loewner ordering. We use such a Loewner ordering of covariance matrices to show that the post-Sparcle covariance matrices capture additional real variability which went unaccounted for in the pre-Sparcle covariance matrices.

1. 11 VIZGEN MERFISH mouse full brain

We demonstrated Sparcle on FoV #75 of the mouse full brain (available from the Vizgen data release program (31)). There were 82 cells and 418 genes. There were 44,000 mRNA within cells and 55,000 dangling mRNA in this FoV. The scRNA-seq data was the same as was used in the MERFISH data. In **Fig. S11**, we see the expression of canonical Vizgen genes (rows) within each cluster (columns) pre- (**a**) and post-Sparcle (**b**), arranged by hierarchical clustering of the pre- and post-Sparcle matrices, respectively. Even in this limited setting of using only one FoV, Sparcle is able to extract clusters (**Fig. S11b**) such as Mature Oligo, Immature Oligo, astrocytes and endothelial cells which were not well defined in the pre-Sparcle matrix (**Fig. S11a**), and Sparcle improves the cell-type specificity of canonical gene assignment for most cell types than before using Sparcle.

Note that there are clusters annotated as 'Mix’ in either the pre- and post-Sparcle matrices consist of both neuronal and non-neuronal cells. There are 5 such ‘mix’ clusters in the pre-Sparcle setting and 3 in the post-Sparcle setting. These ‘Mix’ clusters would be subject to further refinement by combining more FoVs and under the ‘global clustering’ setting of Sparcle.

1. Availability of data and materials

- MERFISH:
  - MERFISH barcode and genes and paired scRNAseq data were obtained from here: <https://www.ncbi.nlm.nih.gov/geo/query/acc.cgi?acc=GSE113576>
  - MERFISH data and corresponding scRNA-seq data can be found at ([11](#_ENREF_11)).
  - FoVs were obtained from the authors (Moffitt et al (11)) directly
  - There are 400 FoVs, each ~3GB and 4.1 Megapixels in size. There are 31,241 cells across the 400 images of which 2.7 million mRNA transcripts fall within cells and 2.8 million are dangling mRNA.
  - The matching scRNA-seq data has 31,299 cells and 27,998 genes.
  - Sparcle_ver_1.ipynb is the Sparcle code (parallelized) to handle the MERFISH FoVs
- smFISH VISp data can be found here:
  - <https://zenodo.org/record/3478502#.YGSMi68zZ3g>. Link courtesy from SSAM (18)
  - We utilise a single image that was stitched and provided by Brian Long from the Allen Brain Institute. The image is that of the primary visual cortex (VISp) region of an adult mouse brain.
  - There are 3500 cells and 22 genes. There were 1074,000 mRNA within this image of which we subsample 250,000 mRNA of which 154,000 mRNA are within cells and 95,000 are dangling mRNA that is used by Sparcle.
  - The matching scRNA-seq data had 43498 cells and 45,000 genes and is available at ([17](#_ENREF_17)).
- STARmap:
  - We utilized data from the 160-gene light experiment (30)
  - In this example we used the count matrix and tables from "visual_160/20180410-BY3_1kgenes" dataset. <https://www.starmapresources.com/data/> -> <https://www.dropbox.com/sh/f7ebheru1lbz91s/AAC-QLN6MqkbrwY6XzpRj-soa/visual_160?dl=0&subfolder_nav_tracking=1> -> 20171120_BF4_light.
  - This had 931 cells, and 511,000 total mRNA and 75,000 unassigned mRNA.
  - Single-cell rnaseq data (32): <https://github.com/AllenInstitute/tasic2016data>.This matching single-cell RNA seq data consisted of 1809 cells and 24057 genes.
- ISS:
  - Data and visualization code (33)
  - Slice used: CA1DapiBoundaries_4-3_right.tif from <https://figshare.com/s/88a0fc8157aca0c6f0e8?file=13160426>
  - This slide had 2024 cells and 92 genes. There were 72,000 mRNA in total of which 11,000 were identified as dangling mRNA.
  - Dimensions of the paired single-cell RNA seq data are 5100 cells and 27998 genes.
- Vizgen FoV 75:
  - We have used data from the recent ‘Vizgen Data release program’ (31)
  - There were 82 cells and 418 genes. There were 44,000 mRNA within cells and 55,000 dangling mRNA in this FoV.

The open-source, platform independent software implementation of Sparcle is available on Github: <https://github.com/sandhya212/Sparcle_for_spot_reassignments>

Code is written in Python 3 and is parallelized to process multiple FoVs during each iteration. We have also developed code that is necessary to reconstruct each FoV from the stage coordinates to image coordinates, plot the cell segment boundaries obtained from the segmentation, and plot the mRNA within cells and dangling mRNAs.

**Supplementary Figures**

**Figure S1:** Graphical abstract for Sparcle. Sparcle iteratively recovers dangling mRNA transcripts. **a.** A MERFISH exemplar FoV showing DAPI channel with cell segments (red lines) and transcripts (points). **b.** A zoomed-in section showing neuronal and non neuronal cells with dangling mRNAs. The neuron at the center shows partial cell segmentation of the nucleus (dark brown region) which contains 4 mRNA transcripts (in pink and green) accounting for 2 genes. The dangling mRNAs present outside the cell segment are completely ignored by current computational downstream approaches. Dotted lines between dangling mRNAs and cells denote potential mRNA to cell assignments. **c.** A further zoomed-in section shows nuclear mRNA as dots as dangling mRNA as crosses. Colors represent one of the 140 genes. Also shown is a mockcell (blue circle) centered at a dangling mRNA (pink cross). **d.** A count matrix of genes x cells is created using cell segments from all FoVs. This is clustered to give a set of cell types along with cluster moments. **e.** Sparcle builds a weighted mockcell for each dangling mRNA and assigns the mockcell to the nearest cell sharing the same cluster as that of the mockcell, using MLE. **f.** The count matrix is updated for cells and relevant genes based on the newly-assigned dangling mRNAs, the count matrix is re-clustered and this process iterates for a fixed number of iterations.

**Figure S2:** Sparcle improves cell type assignment from MERFISH data. **a**$\boldsymbol{.}$ (i) t-SNE projections of MERFISH image data from the mouse hypothalamus optic region comprising 400 fields of view (FoV) with ~30,000 cells total, 140 genes, with matching scRNA-seq data in Moffitt et al. (11) showing diversity in cell type proportions. (ii) Percentage of cells in the paired scRNA-seq data (orange bars), MERFISH (blue bars) and MERFISH with Sparcle (pink bars). Post-Sparcle cell type assignment proportions are in closer accordance with the scRNA-seq proportions, especially for astrocytes, endothelial cells, mature and immature oligodendrocytes, and excitatory and inhibitory neuronal cell types, demonstrating that Sparcle can correct counts per cell type based on the dangling mRNA assignments. **b.** Cluster-cluster covariance (Gramian) matrices of MERFISH (left), MERFISH with Sparcle (center) and scRNA-seq (right) indicate that Sparcle-treated count matrices are more similar to scRNA-seq (pre-Sparcle vs. scRNA-seq median χ² (2, N = ~61,000) = 0.26, p = 0.31, Frobenius norm = 15.18; post-Sparcle vs. scRNA-seq median χ² (2, N = ~61,000) = 0.95, p = 0.62, Frobenius norm = 2.34). **c**. Cluster-cluster cross covariance matrices between scRNA-seq and MERFISH clusters pre- and post-Sparcle indicate stronger within-cluster variances (dominant diagonal) after Sparcle, especially for non-neuronal cell types.

**Figure S3:** Sparcle refines clusters in MERFISH data. **a.** We constructed a pre-Sparcle MERFISH matrix with 100 randomly sampled excitatory cells stacked over 100 inhibitory cells and a post-Sparcle matrix with the same cells, then computed Pearson correlations with a similarly constructed scRNA-seq matrix of 100 random cells per cell type. Pearson correlation matrices indicate greater covariance across diagonal blocks (defined red zones) between scRNA-seq and post-Sparcle MERFISH data, for both excitatory and inhibitory neuronal cell types. **b.** Similar analysis reveals the same trends for astrocytes, endothelial and ependymal cells (NN set 1) and immature and mature oligodendrocytes, microglia and mural cells (NN set 2).

**Figure S4:** Sparcle generalizes to smFISH data. **a**. Single smFISH FoV of mouse visual cortex containing 3500 cells and 250,000 randomly subsampled mRNAs; of 93,000 dangling transcripts, 3300 are shown for visual clarity. Cells and connections to dangling mRNAs from 22 genes are colored based on matching scRNA-seq clusters. Inset, magnification. **b**. Cluster-cluster covariance (Gramian) matrices between (i) smFISH, (ii) smFISH with Sparcle and (iii) scRNA-seq indicate that Sparcle-treated count matrices are more similar to scRNA-seq (Box’s M test for pre-Sparcle vs. scRNA-seq median χ² (2, N = 3500) = 0.36, p = 0.48, Frobenius norm = 9.42; Box’s M test for post-Sparcle vs. scRNA-seq median χ² (2, N = 3500) = 0.92, p = 0.78, Frobenius norm = 1.83). **c**. Cluster-cluster cross covariance matrices between scRNA-seq and MERFISH clusters pre- and post-Sparcle indicate stronger within-cluster variances (dominant diagonal) after Sparcle. **d**. Expression of 22 smFISH genes (rows) within each cluster (columns) pre- and post-Sparcle, arranged by hierarchical clustering of the pre- and post-Sparcle matrices, respectively. Sparcle generates clearer blocks of gene expression per cluster and improves assignments for all four cell types. Color scale in all panels represents z-scored values of log-normalized expression per cluster.

**Figure S5:** Weighted Mockcell creation. A MERFISH FoV showing spatial allocation of 134 genes in a section of the mouse hypothalamus optic region (11). Dots represent mRNA within cell segmentations, colored by cell cluster assignment. Crosses represent unassigned (dangling) mRNA. Inset, a zoomed-in region showing mock cell (blue circle), centred at a dangling mRNA (pink cross). All mRNA (dots and crosses) within the circle will contribute to that mock cell, weighted inversely to the distance from the central mRNA.

**Figure S6**: Mockcell creation. **a**. A MERFISH image shown in image coordinates after perspective projection from stage coordinates. This projection allows the x and y axis to start from (0,0) and enables the calculation of Euclidean distances between cell centroids and mRNA transcripts. We use this to estimate the radius r for a mockcell body. For example, we draw a bounding box around cell A and cell B and observe that its radius is around 80 pixels. This is used as a proxy for the mockcell body creation. **b**. The same MERFISH image recreated in Python using cell segmentation coordinates to outline the neuronal cells. The cells are colored based on their cell type. A dangling mRNA is shown as a black cross with red crosses, traced within a circle of radius 150 pixels to include the neuronal processes, identifying those mRNA that will contribute to its mockcell (i.e. including both the cell body and neuronal processes).

**Figure S7**: Allen smFISH data and matching scRNA-seq data. **a**. Single smFISH FoV of mouse visual cortex containing 3500 cells and 250,000 randomly subsampled mRNAs; of 93,000 dangling transcripts, 3300 are shown for visual clarity. Cells and dangling mRNAs are colored based on matching scRNA-seq clusters. Inset, magnification with dangling mRNAs (+ symbols). The dangling mRNAs account for 22 smFISH genes: *Alcam, Chodl, Cux2, Fezf2, Foxp2, Gad2, Galnt14, Grin3a, Kcnip4, Kcnk2, Lhx6, Mpped1, Parm1, Pde1a, Prox1, Pvalb, Rorb, Satb2, Sema3e, Sez6, Sv2c, Thsd7a.* **b**. tSNE projection showing proportions of the four hierarchical clusters corresponding to endothelial (Endo), excitatory (Ex), glial (Glia) and inhibitory (Inh) cell types of the matching scRNA-seq data, which comprises 43,498 cells and 45,000 genes.

**Figure S8**: Percentage of mRNA recovery across the 4 dominant cell types using Sparcle in the Allen smFISH data. We subsample 250,000 mRNAs from a total of 1,074,000 within this image; 154,000 mRNAs (61.8%) are within cells and 95,000 (38.2%) are dangling. Cell type proportions of within-cell mRNA in the smFISH data are in blue (endothelial 8.1%, excitatory 22.7%, glial 11.8%, inhibitory 19.3%), and additional dangling transcripts assigned by Sparcle are in pink, comprising 32.2% of the total mRNA (endothelial 5.5%, excitatory 11.25%, glial 6.1%, inhibitory 9.3%) across three iterations; only 6% of the total mRNA remains unassigned.

**Figure S9:** Sparcle generalizes to brain interstitial system (ISS) CA1 Hippocampus data (slice DAPI_4-3.jpg from https://figshare.com/s/88a0fc8157aca0c6f0e8?file=13160492). (**a.,b.**) Cluster-cluster cross covariance matrices between published scRNA-seq (33) and ISS clusters pre- (**a**) and post-Sparcle (**b**). Note there is clearer separation of interneuron-selective (IS) cell types (IS1, IS3 and IS2) without any mixing amongst the cell types post-Sparcle. Further, the trilaminar, radiatum retrohip and Cck+ (Cck Cxcl14+) clusters are also well separated post-Sparcle. These results further indicate that Sparcle can be used as a count matrix refinement tool after pciSeq is applied on the ISS data.

Cell types shown here are O/LM (oriens/lacunosum-moleculare); Axo (axo-axonic); Hippo (Hippocamposeptal); Bistrat (Bistratified); Basket; MGE (medial ganglionic eminence neurogliaform); CGE (caudal ganglionic eminence); Trilam (Trilaminar); Radiatum (Radiatum retrohip); Cck+ (Cck Cxcl14+); Cck- (Cck Cxcl14-);IS, (interneuron-selective 1/2/3)

Data dimensions:

- pciSeq data: 92 genes, 2010 cells, mRNA within cells ~55K, dangling mRNA ~10K
- single-cell RNA seq data: 5712 genes, 27998 cells

**Figure S10:** Sparcle generalizes to STARmap 160-gene light data of the mouse visual cortex. (**a.,b.**) Expression of canonical STARmap genes (rows) within each cluster (columns) pre- (**a**) and post-Sparcle (**b**), arranged by hierarchical clustering of the pre- and post-Sparcle matrices, respectively. Sparcle generates clearer blocks of gene expression per cluster and improves assignments for all the cell types. Color scale in all panels represents z-scored values of log-normalized expression per cluster. (**c.,d.**) Comparing Cluster-cluster cross covariance matrices between published scRNA-seq (32) and STARmap clusters pre- (**c**) and post-Sparcle (**d**) indicate stronger within-cluster variances (dominant diagonal) after Sparcle. Note there is a clearer separation of inhibitory neuronal cell types (Vip, Sst, Pvalb) without any mixing with excitatory neuronal clusters (eL2/3, eL4, eL5, and eL6) post-Sparcle.

Data dimensions:

- STARmap data: 160 genes, 931 cells, mRNA within cells ~511K, dangling mRNA ~75K
- single-cell RNA seq data: 24K genes, 1809 cells

**Figure S11:** Sparcle run on Vizgen Fov#75: Expression of canonical Vizgen genes (rows) within each cluster (columns) pre- (**a**) and post-Sparcle (**b**), arranged by hierarchical clustering of the pre- and post-Sparcle matrices, respectively. Color scale in all panels represents z-scored values of log-normalized expression per cluster. Even though using only one FoV would not capture the entire refinement of cell types through Sparcle, we show that even in this limited setting Sparcle is able to extract clusters (in **b**) such as Mature Oligo, Immature Oligo, astrocytes and endothelial cells which were not well defined in the pre-Sparcle matrix (**a**). Clusters are annotated when their Pearson correlation coefficient is above 1.9 (indicated as dark red squares in the matrices). Clusters annotated as 'Mix’ in either the pre- and post-Sparcle matrices consist of both neuronal and non-neuronal cells. There are 5 such ‘mix’ clusters in the pre-Sparcle setting and 3 in the post-Sparcle setting. These ‘Mix’ clusters would be subject to further refinement by combining more FoVs and under the ‘global clustering’ setting of Sparcle.

Data dimensions:

- VIZGEN FoV 75 data: 418 genes, 82 cells, mRNA within cells ~44K, dangling mRNA ~55K

**Figure S12:** Sparcle’s performance on finer cell (sub)type resolutions for smFISH. We follow the cell type annotations from SSAM (18) and scRNA-seq data (17). The figures indicate that Sparcle improves cell types (for e,g. Lamp5, Sst, L4IT1,Oligo/VLMC). **(a.,b.**) Cluster-cluster cross covariance matrices between published scRNA-seq data (32) and smFISH clusters pre- (**a**) and post-Sparcle (**b**). Note that in the post-Sparcle setting: 1. There is a clearer separation of Lamp5, Sst clusters. 2. There is no mixing of L2/3 IT1 with L6 IT 1/2 and Oligo/VLMC. 3. There is no mixing of L4 IT1 with Oligo/VLMC. (**c.,d.**) Expression of the 22 smFISH genes (rows) within each cluster (columns) pre- (**c**) and post-Sparcle (**d**), arranged by hierarchical clustering of the pre- and post-Sparcle matrices, respectively. Color scale in all panels represents z-scored values of log-normalized expression per cluster. Note that in the post-Sparcle setting, 1. RorB and Cux2- expressed excitatory cells retain their individual clusters and 2. Cux2 is better refined for L2/3 IT 1 whereas in the pre-Sparcle setting, Cux2 was spread across L2/3 IT 1, Oligo/VLMC and L4 IT 1.  The clusters are labelled following the convention in the SSAM paper Figure 5A (18).

Data dimensions:

- Allen smFISH data: 3500 cells, and 250,000 randomly subsampled mRNAs; of which 93,000 dangling transcripts
- single-cell RNA seq data: 45K genes, 43,498 cells

References

1. Chen KH, Boettiger AN, Moffitt JR, Wang S, Zhuang X. RNA imaging. Spatially resolved, highly multiplexed RNA profiling in single cells. Science. 2015;348(6233).

2. Codeluppi S, Borm L, Zeisel A, La Manno G, van Lunteren J, Svensson C, et al. Spatial organization of the somatosensory cortex revealed by osmFISH. Nature Methods. 2018;15(11):932-5.

3. Eng CL, Lawson M, Zhu Q, Dries R, Koulena N, Takei Y, et al. Transcriptome-scale super-resolved imaging in tissues by RNA seqFISH. Nature. 2019;568(7751):235-9.

4. Long X, Colonell J, Wong A, Singer R, Lionnet T. Quantitative mRNA imaging throughout the entire Drosophila brain. Nature Methods. 2017;14(7):703-6.

5. Lubeck E, Coskun A, Zhiyentayev T, Ahmad M, Cai L. Single-cell in situ RNA profiling by sequential hybridization. Nature Methods. 2014;11(4):360-1.

6. Nichterwitz S, Chen G, Aguila BJ, Yilmaz M, Storvall H, Cao M, et al. Laser capture microscopy coupled with Smart-seq2 for precise spatial transcriptomic profiling. Nature Communications. 2016;7(12139).

7. Marx V. Method of the Year: spatially resolved transcriptomics. Nature Methods. 2021;18(219).

8. Asp M, Bergenstråhle J, Lundeberg J. Spatially Resolved Transcriptomes—Next Generation Tools for Tissue Exploration. Wiley Periodicals. 2020;42(10).

9. Battich N, Stoeger T, Pelkmans L. Image-based transcriptomics in thousands of single human cells at single-molecule resolution. Nature Methods. 2013;10(11):1127-33.

10. Shah S, Lubeck E, Zhou W, Cai L. In situ transcription profiling of single cells reveals spatial organization of cells in the mouse hippocampus. Neuron. 2016;92(2):342-57.

11. Moffitt J, Bambah-Mukku D, Eichhorn S, Vaughn E, Shekhar K, Perez J, et al. Molecular, spatial, and functional single-cell profiling of the hypothalamic preoptic region. Science. 2018;362(6416).

12. Braiki M, Benzinou A, Nasreddine K, Hymery N. Automatic Human Dendritic Cells Segmentation Using K-Means Clustering and Chan-Vese Active Contour Model. Comput Methods Programs Biomed. 2020;195(105520).

13. Neal RM. Markov chain sampling methods for Dirichlet process mixture models. Journal of Computational and Graphical Statistics. 2000;9:249-65.

14. Teh YW, Jordan MI, Beal MJ, Blei DM. Hierarchical dirichlet processes. Journal of the American Statistical Association. 2006;101(476):1556-81.

15. Prabhakaran S, Azizi E, Carr A, Pe'er D. Dirichlet Process Mixture Model for Correcting Technical Variation in Single-Cell Gene Expression Data. Journal of Machine Learning Research (JMLR) Workshop and Conference Proceedings. 2016;48:1070-9.

16. Levine J, Simonds E, Bendall S, Davis K, Amir e-A, Tadmor M, et al. Data-Driven Phenotypic Dissection of AML Reveals Progenitor-like Cells that Correlate with Prognosis. Cell. 2015;162(1):184-97.

17. Allen Brain Map. Cell Types Database: RNA-Seq Data <https://portal.brain-map.org/atlases-and-data/rnaseq#Mouse_Cortex_and_Hip> [

18. Park J, Choi W, Tiesmeyer S, Long B, Borm LE, Garren E, et al. Cell segmentation-free inference of cell types from in situ transcriptomics data. Nature Communications. 2021;12(1), 1-13

19. Petukhov V, Xu RJ, Soldatov RA, Cadinu P, Khodosevich K, Moffitt JR and Kharchenko PV. (2021) Cell segmentation of image-based spatial transcriptomics. Nature Biotechnology. 40(3), 345–354.

20. Greenwald NF, Miller G, Moen E, Kong A, Kagel A, Fullaway CC, et al. Whole-cell segmentation of tissue images with human-level performance using large-scale data

annotation and deep learning. Nature biotechnology, 2022: 40(4), 555–565.

21. Shepard D. A two-dimensional interpolation function for irregularly-spaced data. ACM National Conference. 1968:517-24.

22. Myung IJ. Tutorial on maximum likelihood estimation. Journal of mathematical Psychology. 2003;47(1):90-100.

23. Bindel D, Demmel J, Kahan W, Marques O. On Computing Givens rotations reliably and efficiently. LAPACK Working Note 148. 2001.

24. Bartel D. qr_decomposition 2015. Available from: <https://github.com/danbar/qr_decomposition>.

25. Box GEP. A general distribution theory for a class of likelihood criteria. Biometrika. 1949;36:317-46

26. Hahs-Vaughn D. Applied Multivariate Statistical Concepts: Taylor & Francis; 2016.

27. Glen S. Box’s M Test: Definition [Available from: https://www.statisticshowto.com/boxs-m-test/.

28. Rao CR. Linear Statistical Inference and its Applications. 2 ed: Wiley; 1973.

29. [Qian](https://www.nature.com/articles/s41592-019-0631-4#auth-Xiaoyan-Qian) X, Harris DK, Hauling T, [Nicoloutsopoulos](https://www.nature.com/articles/s41592-019-0631-4#auth-Dimitris-Nicoloutsopoulos) D, [Muñoz-Manchado](https://www.nature.com/articles/s41592-019-0631-4#auth-Ana_B_-Mu_oz_Manchado) BA, [Skene](https://www.nature.com/articles/s41592-019-0631-4#auth-Nathan-Skene) N, [Hjerling-Leffler](https://www.nature.com/articles/s41592-019-0631-4#auth-Jens-Hjerling_Leffler) J, and Nilsson M. Probabilistic cell typing enables fine mapping of closely related cell types in situ. Nature Methods. 2020; 17: 101-6.

30. Wang X, Allen WE, Wright MA, Sylwestrak EL, Samusik N, Vesuna S, Evans K, Liu C, Ramakrishnan C, Liu J, Nolan GP, Bava FA, Deisseroth K. Three-dimensional intact-tissue sequencing of single-cell transcriptional states. Science. 2018; 361(6400)

31. Vizgen MERFISH Mouse Brain Receptor Map: <https://info.vizgen.com/mouse-brain-data>. 2021.

32. Tasic, B., Menon, V., Nguyen, T. N., Kim, T. K., Jarsky, T., Yao, Z., Levi, B., Gray, L. T., Sorensen, S. A., Dolbeare, T., Bertagnolli, D., Goldy, J., Shapovalova, N., Parry, S., Lee, C., Smith, K., Bernard, A., Madisen, L., Sunkin, S. M., Hawrylycz, M., … Zeng, H. (2016). Adult mouse cortical cell taxonomy revealed by single cell transcriptomics. Nature neuroscience, 19(2), 335–346. https://doi.org/10.1038/nn.4216

33. Data and visualization code for ISS/pciSeq: https://colab.research.google.com/github/acycliq/pciSeq/blob/master/notebooks/pciSeq.ipynb
