# Supplementary figures and images for: Sparcle: assigning transcripts to cells in multiplexed images
Source: Bioinform Adv. 2022 Jun 17;2(1):vbac048. doi: 10.1093/bioadv/vbac048 (PMC9710569; doi:10.1093/bioadv/vbac048)

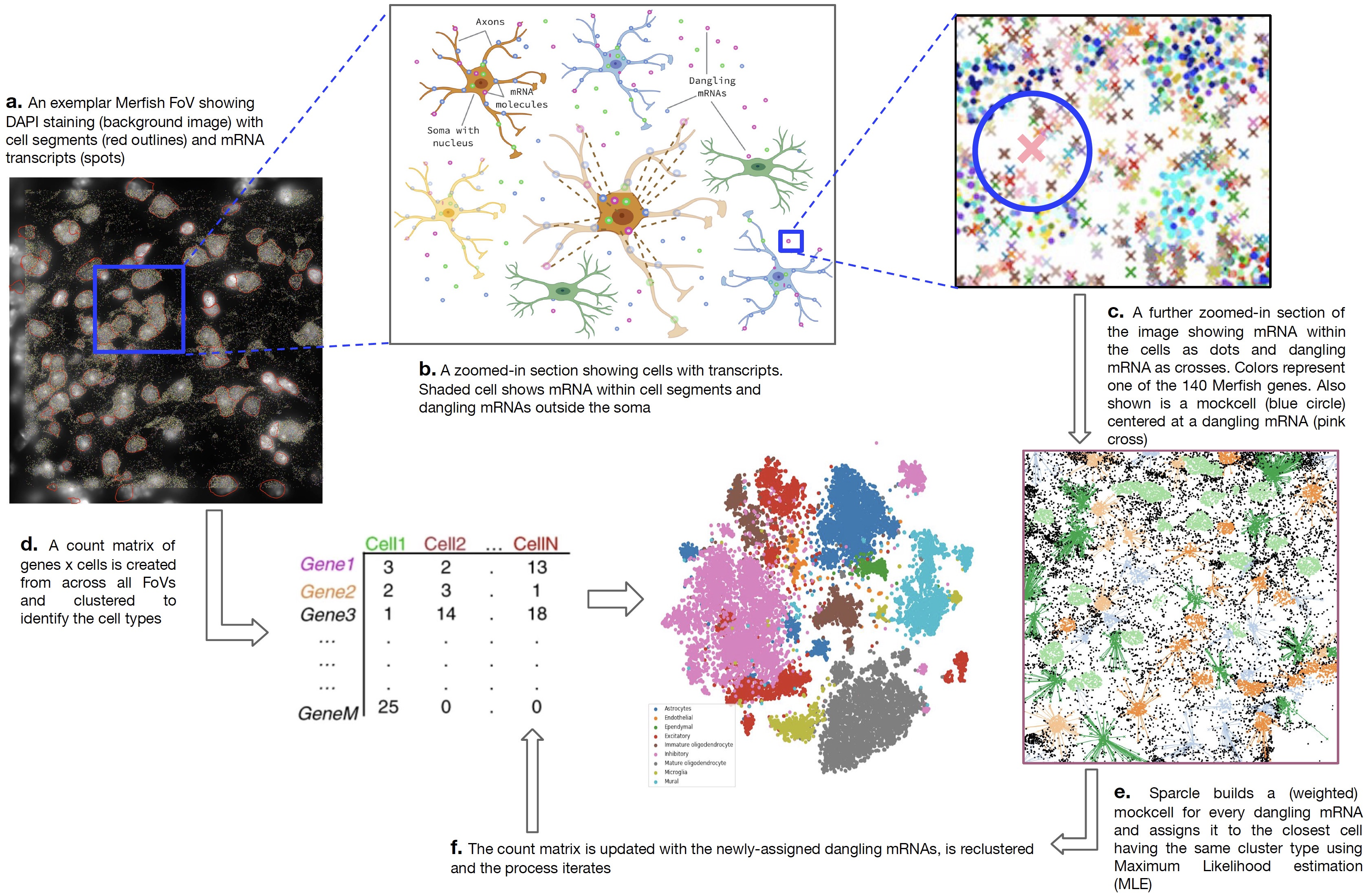

Supplement: vbac048_Supplementary_Data [file vbac048_supplementary_data.zip › Figure S1.tiff]

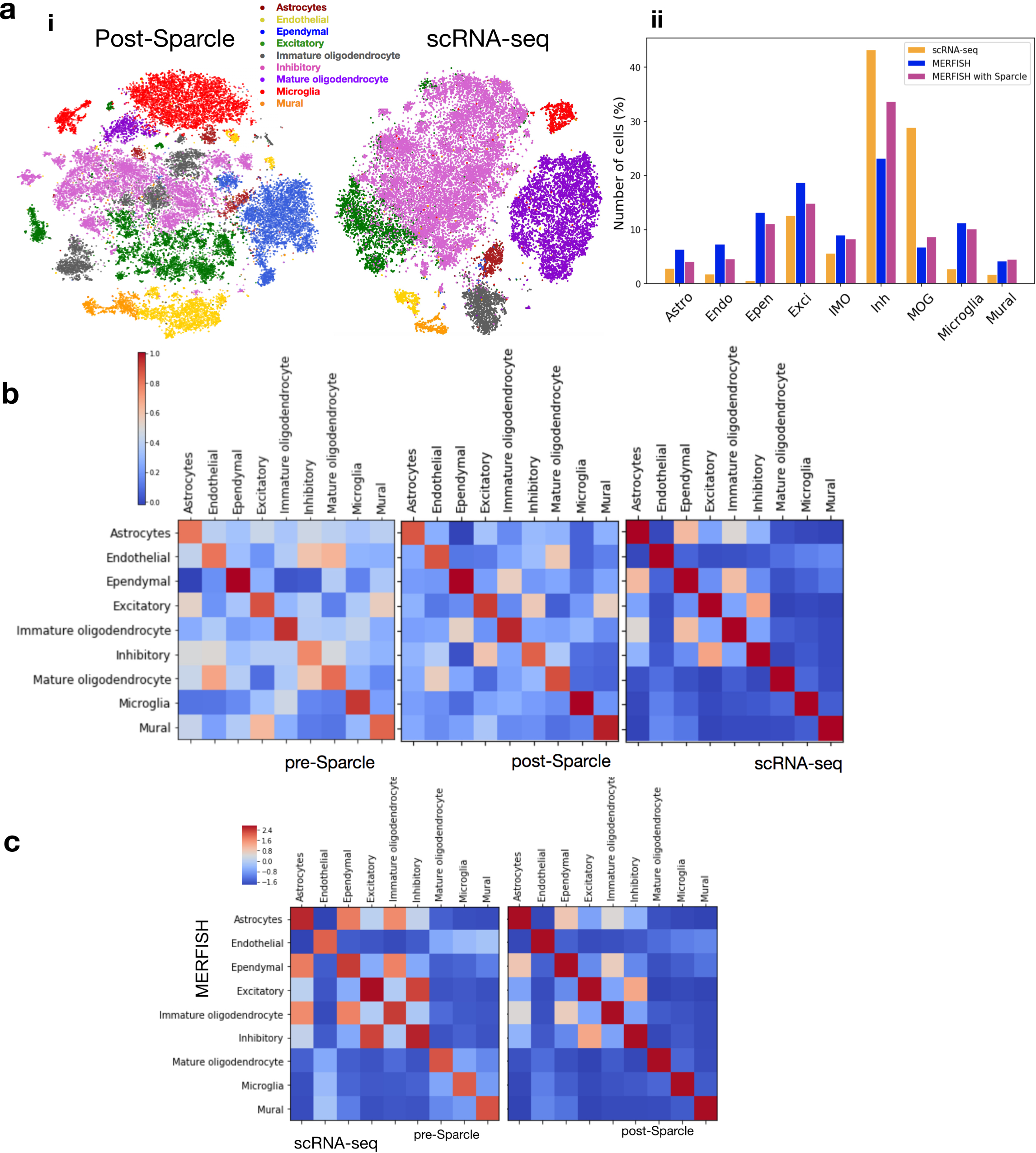

Supplement: vbac048_Supplementary_Data [file vbac048_supplementary_data.zip › Figure S2.tiff]

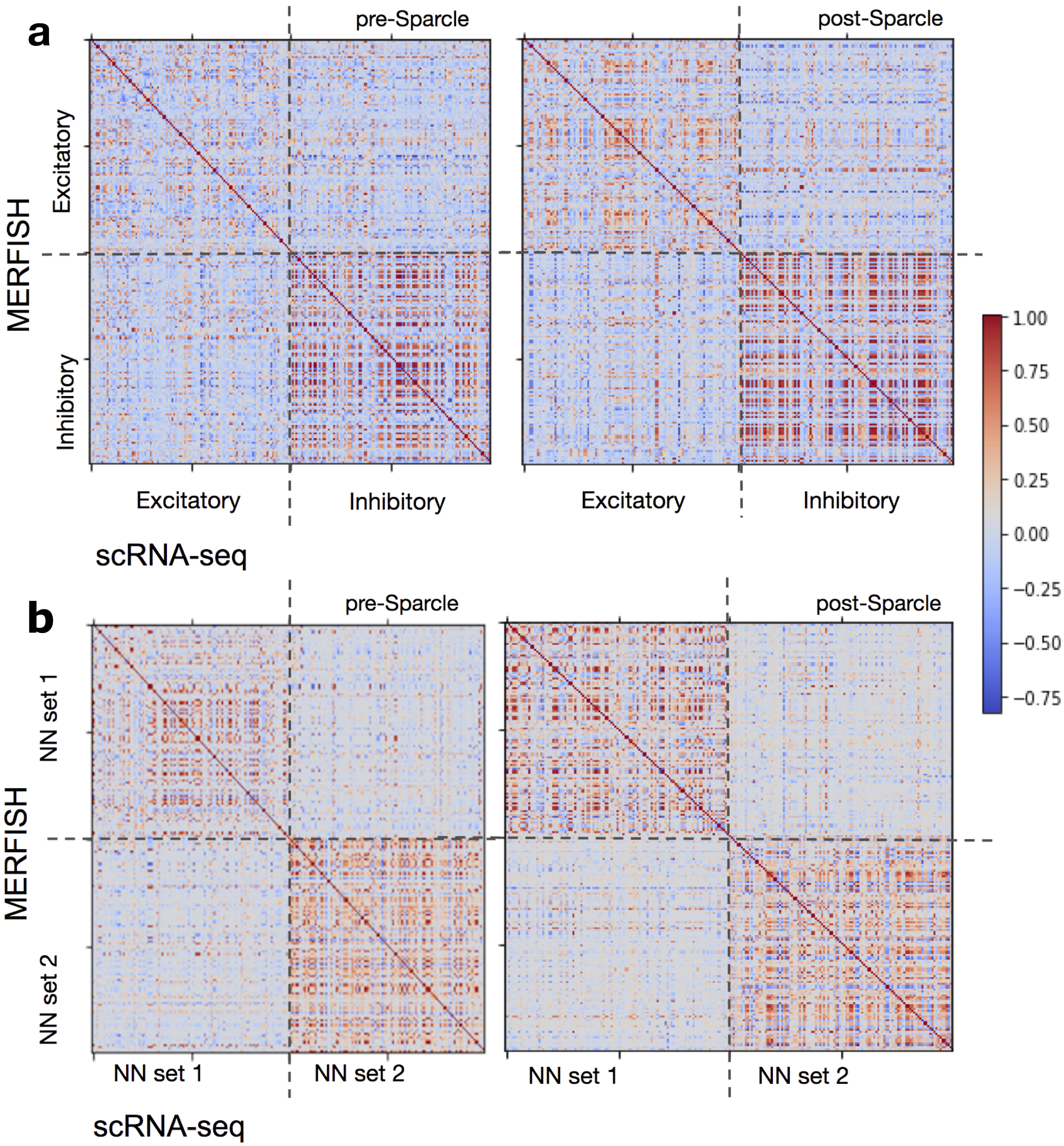

Supplement: vbac048_Supplementary_Data [file vbac048_supplementary_data.zip › Figure S3.tiff]

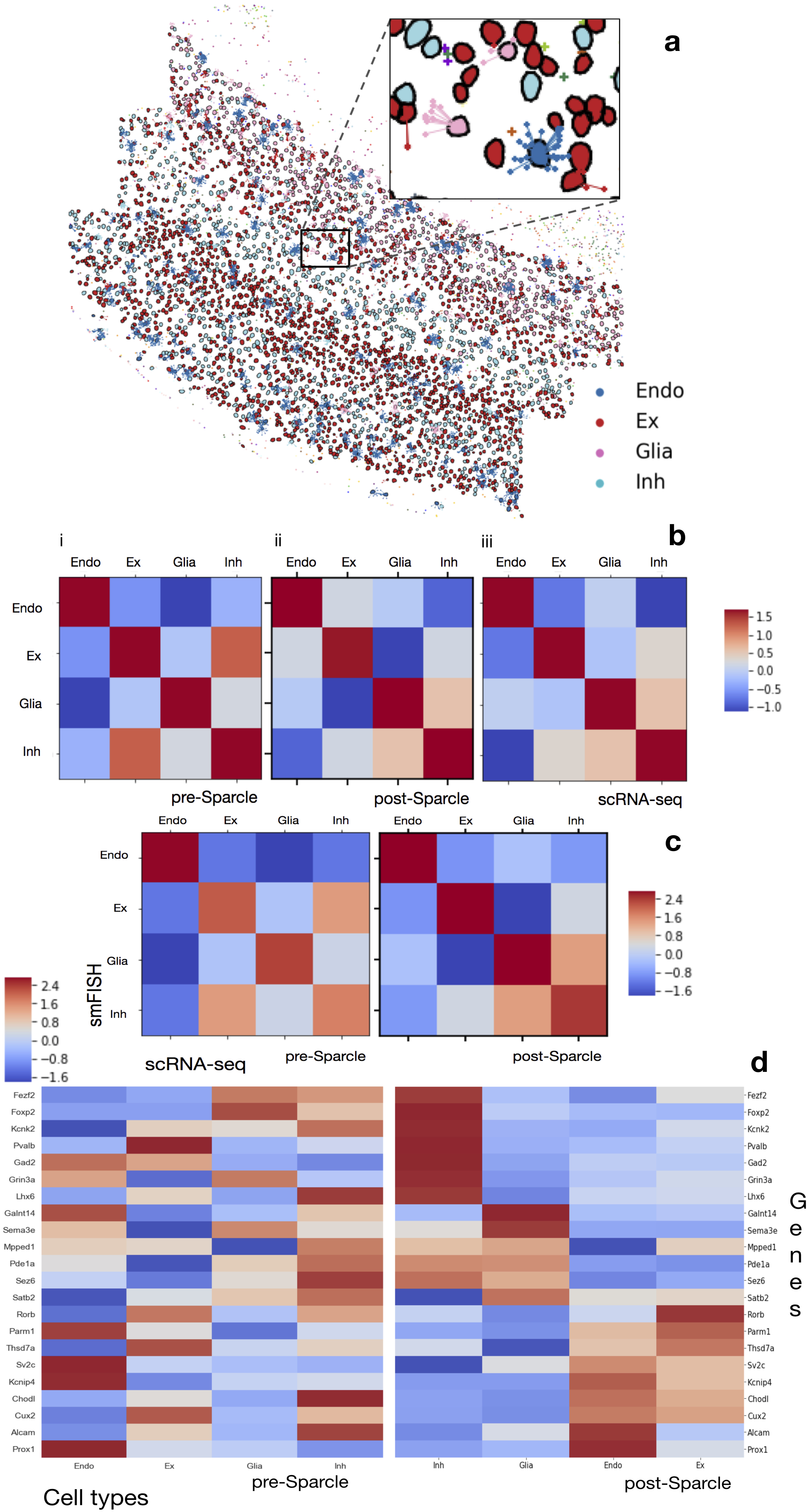

Supplement: vbac048_Supplementary_Data [file vbac048_supplementary_data.zip › Figure S4.tiff]

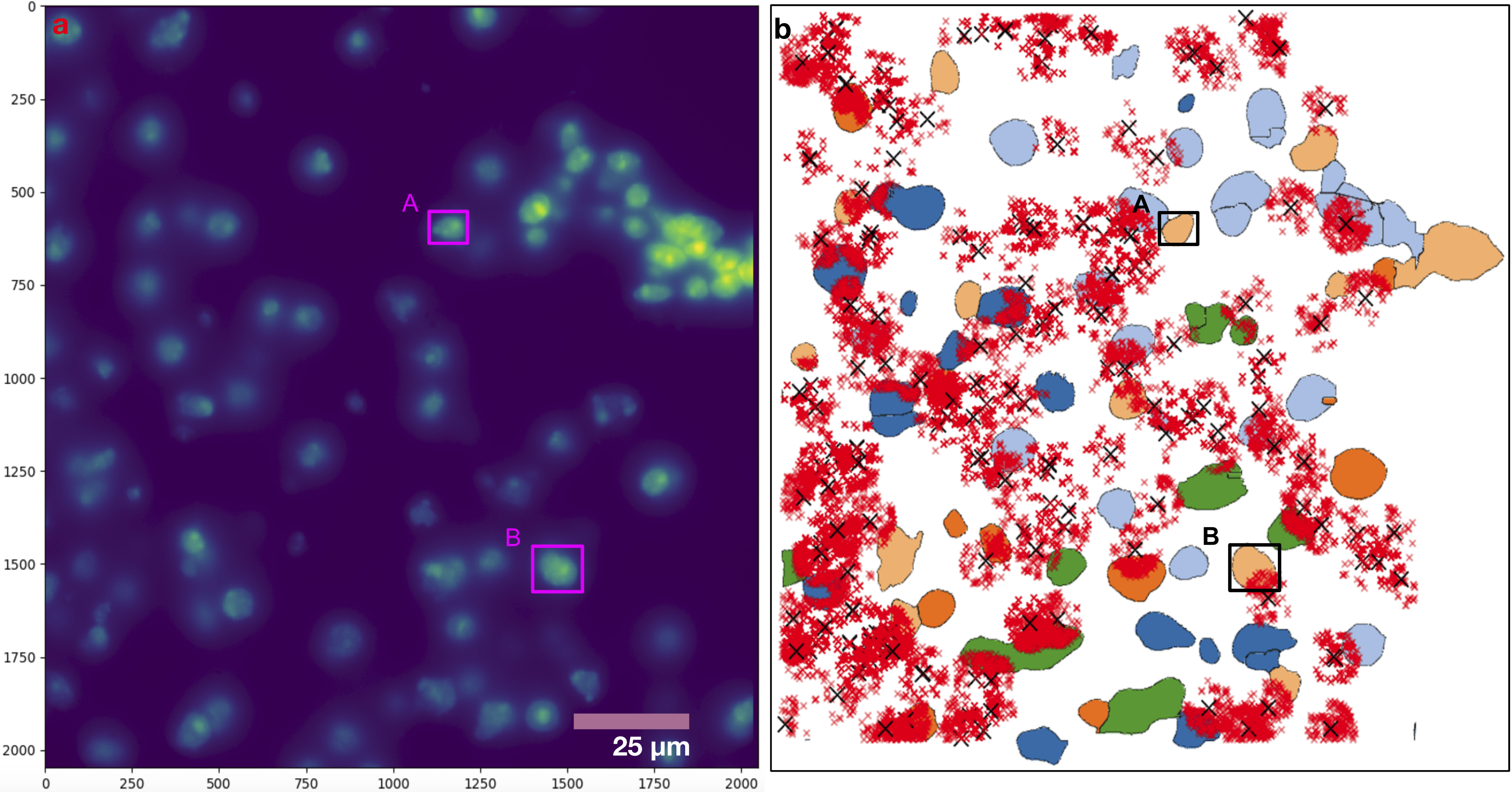

Supplement: vbac048_Supplementary_Data [file vbac048_supplementary_data.zip › Figure S6.tiff]

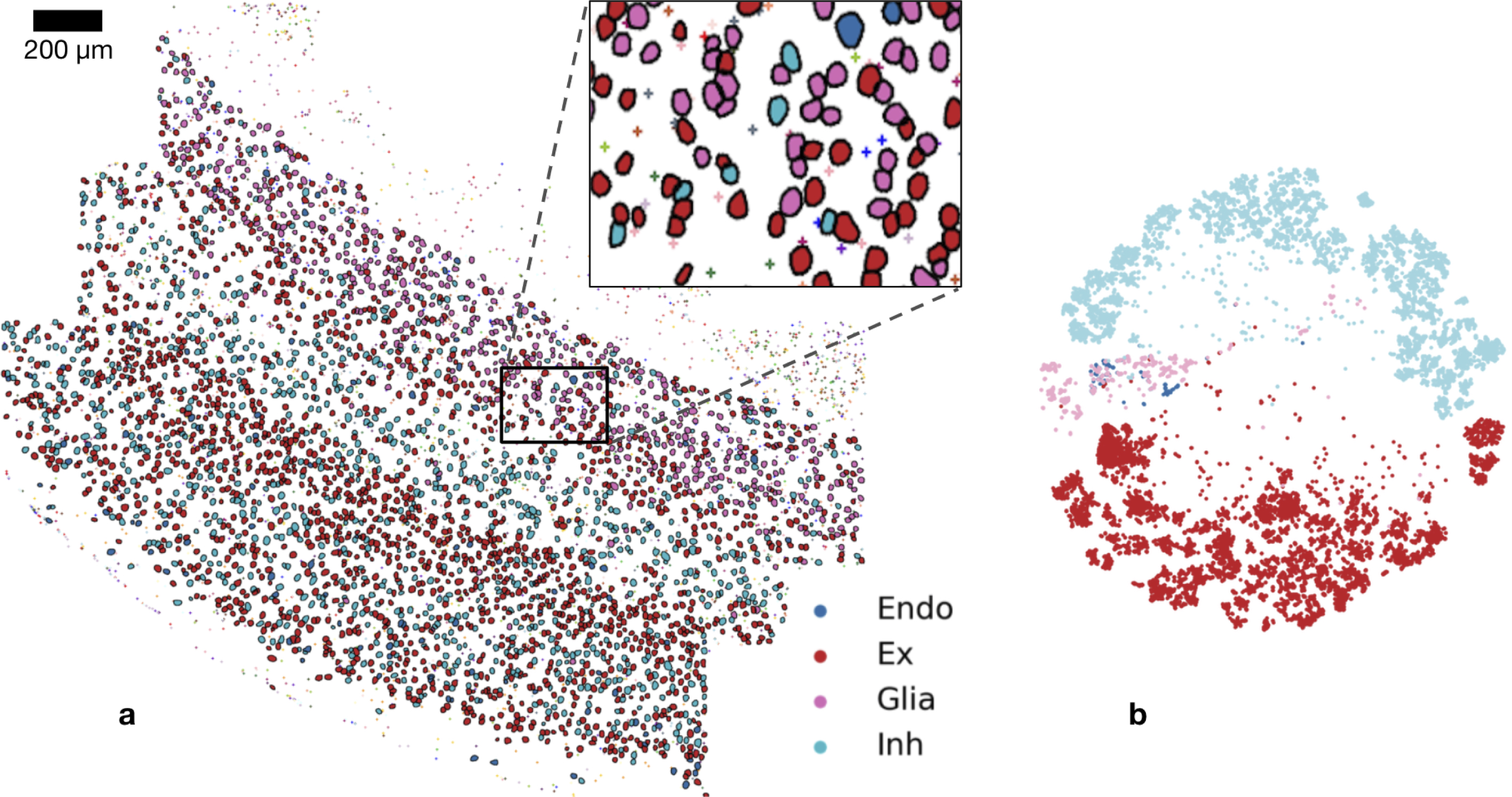

Supplement: vbac048_Supplementary_Data [file vbac048_supplementary_data.zip › Figure S7.tiff]

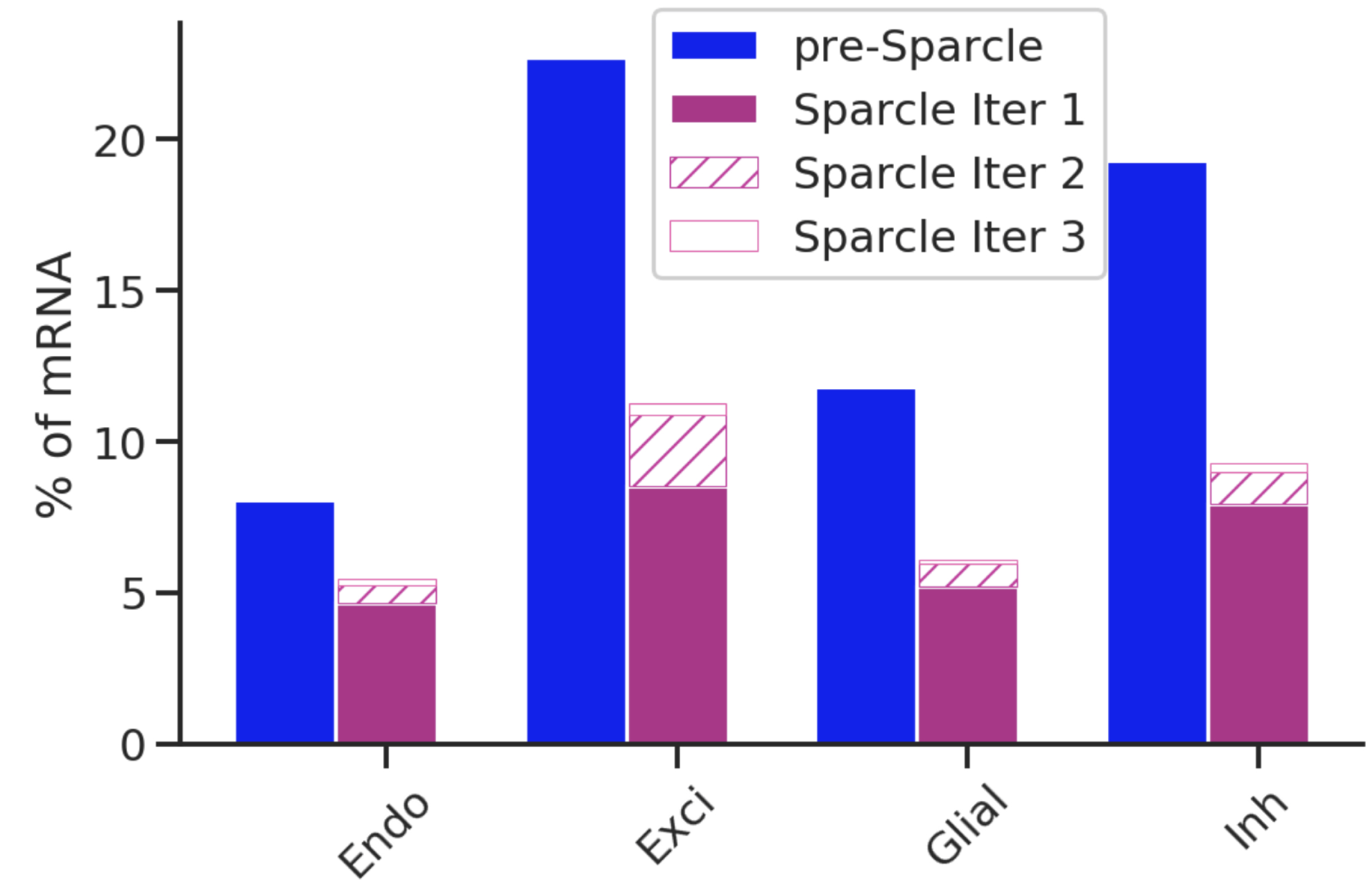

Supplement: vbac048_Supplementary_Data [file vbac048_supplementary_data.zip › Figure S8.tiff]

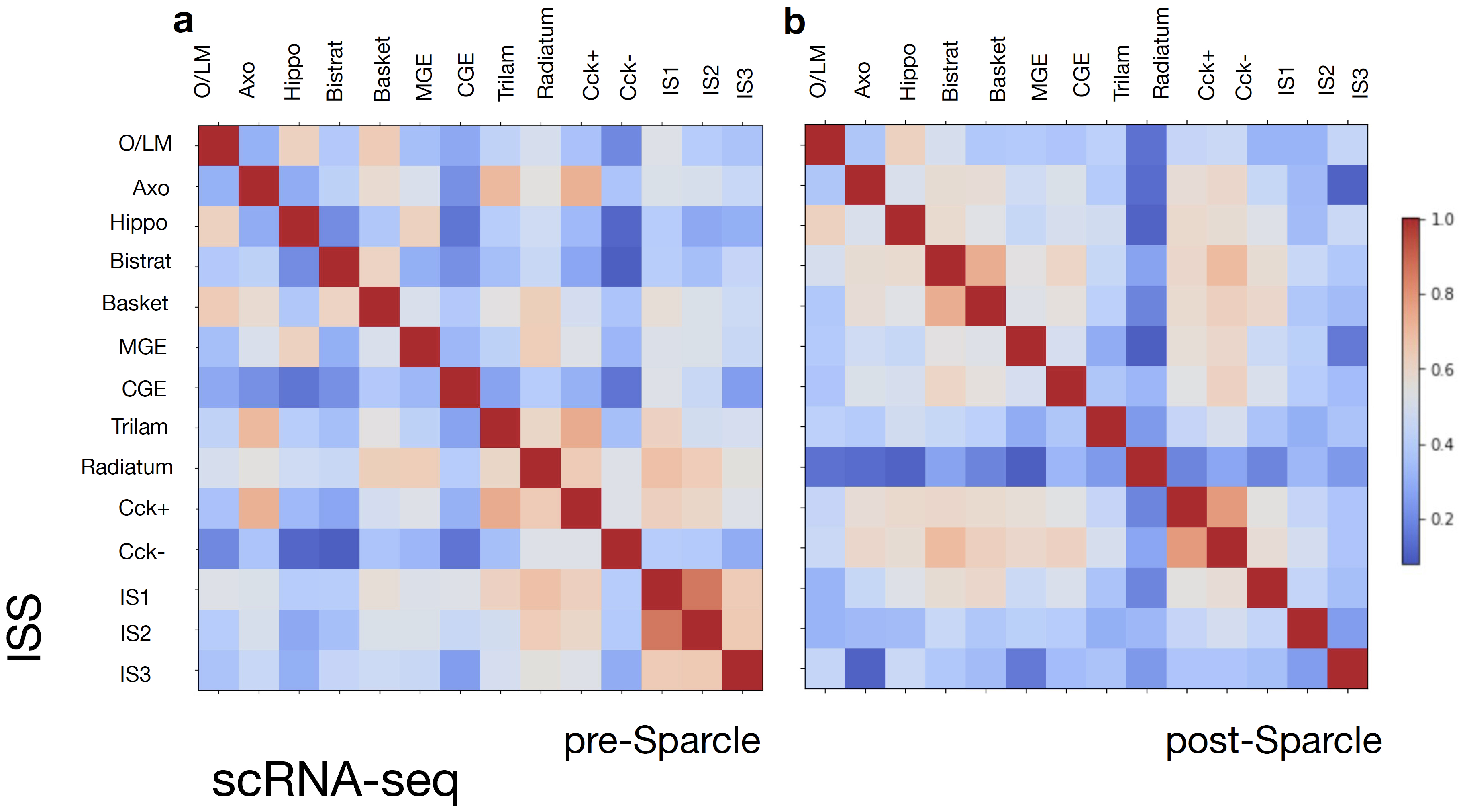

Supplement: vbac048_Supplementary_Data [file vbac048_supplementary_data.zip › Figure S9.tiff]

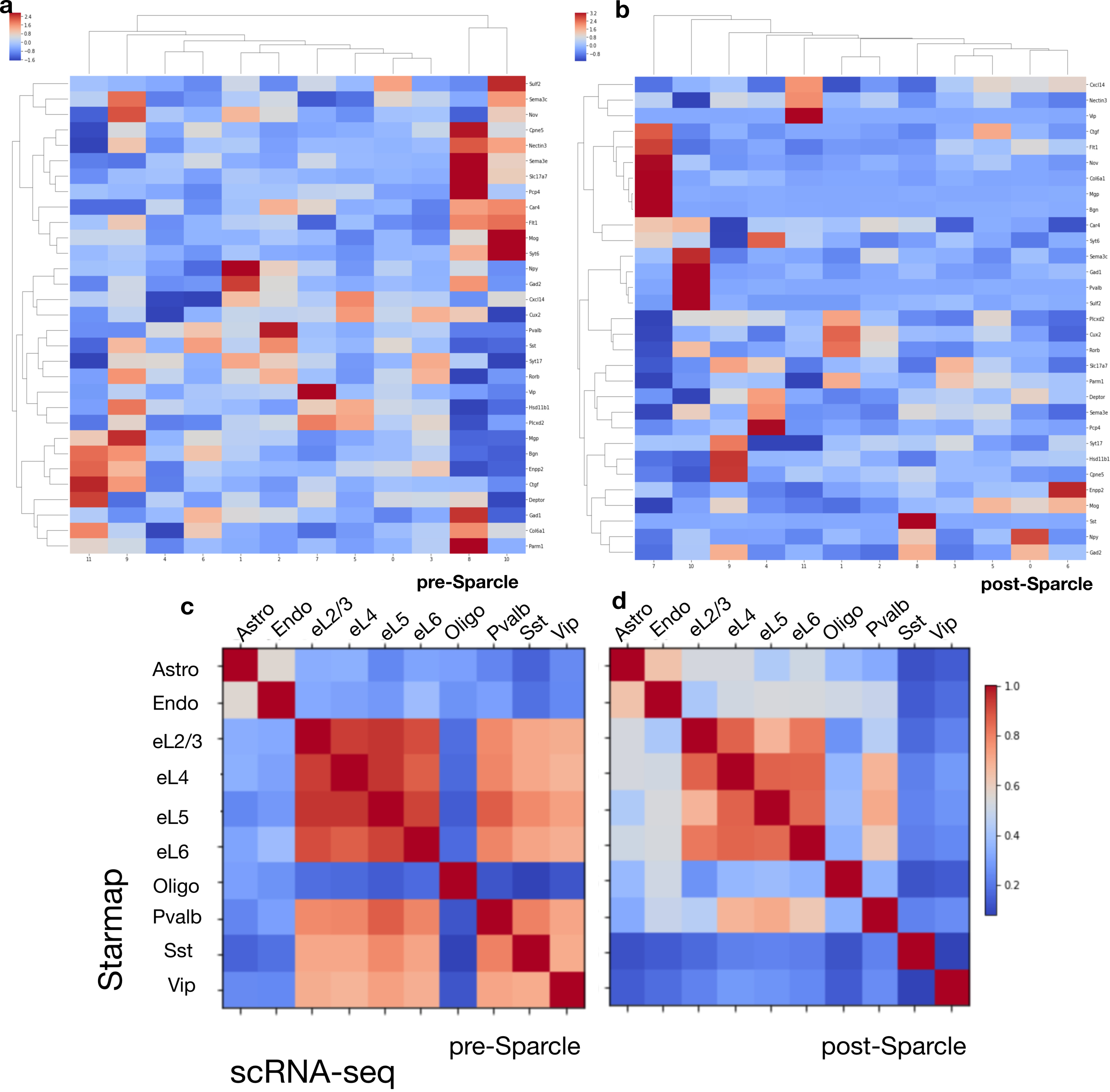

Supplement: vbac048_Supplementary_Data [file vbac048_supplementary_data.zip › Figure S10.tiff]

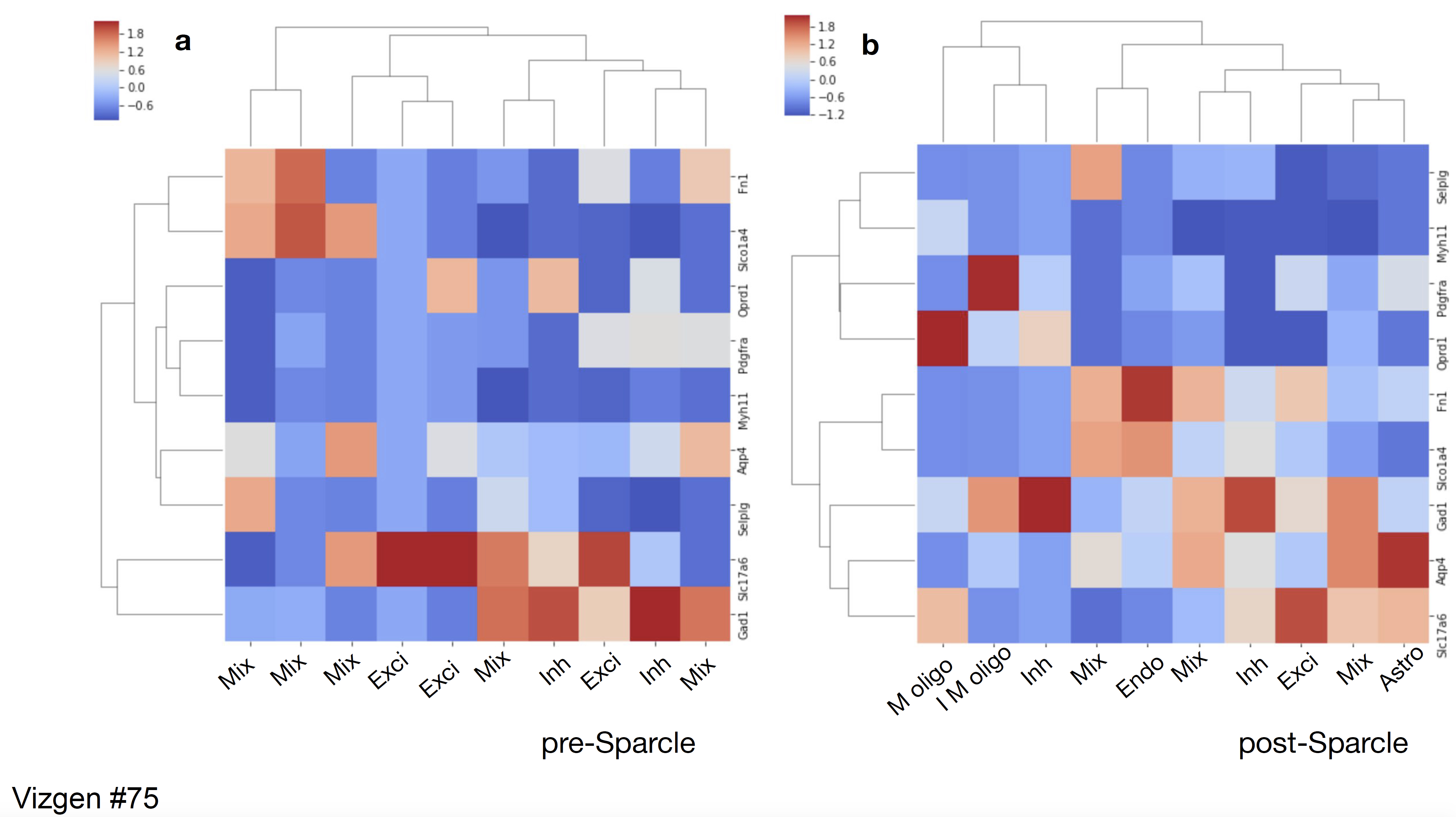

Supplement: vbac048_Supplementary_Data [file vbac048_supplementary_data.zip › Figure S11.tiff]

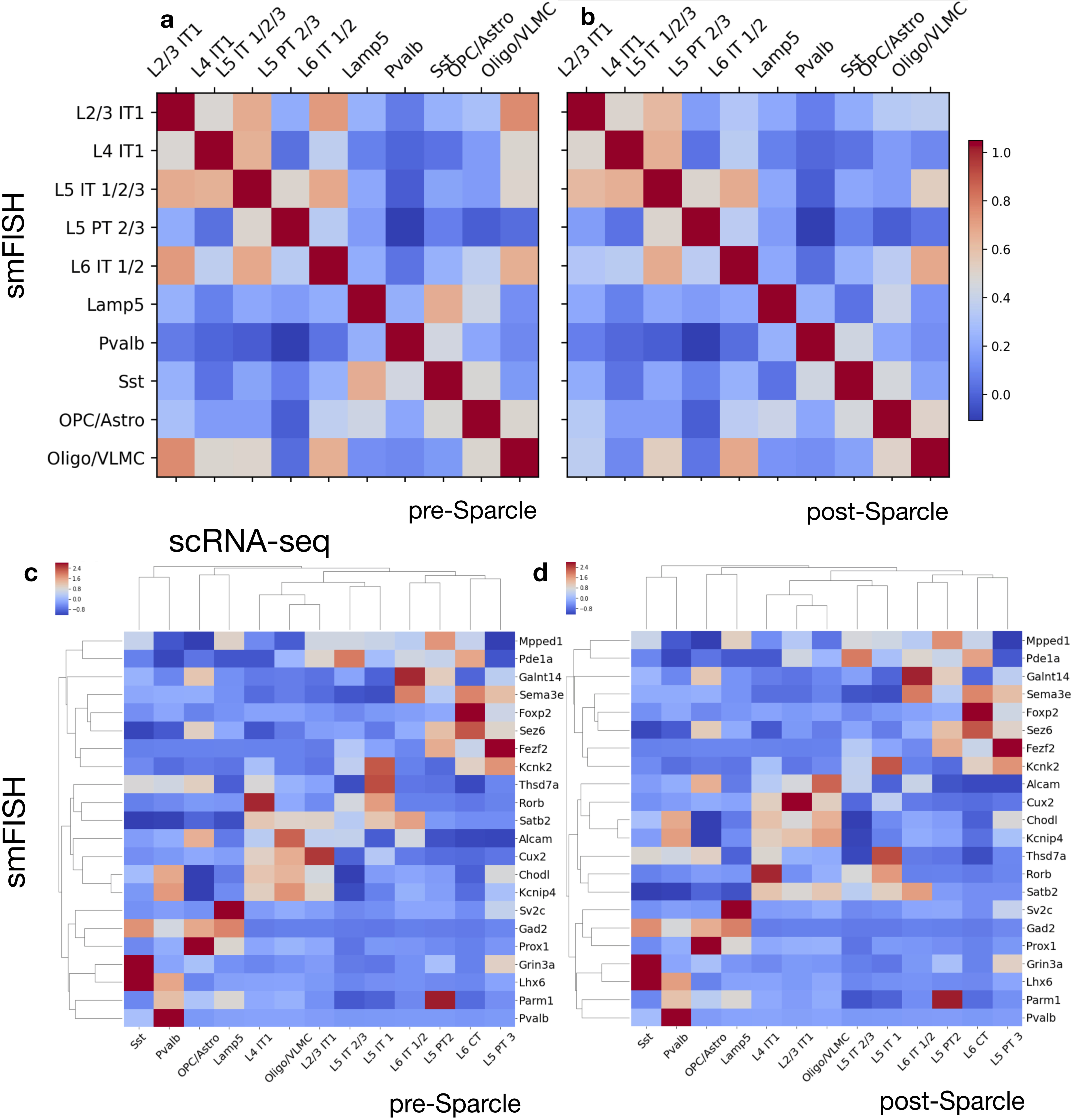

Supplement: vbac048_Supplementary_Data [file vbac048_supplementary_data.zip › Figure S12.tiff]
